# RanBP2/Nup358 enhances miRNA activity by sumoylating Argonautes

**DOI:** 10.1371/journal.pgen.1009378

**Published:** 2021-02-18

**Authors:** Qingtang Shen, Yifan E. Wang, Mathew Truong, Kohila Mahadevan, Jingze J. Wu, Hui Zhang, Jiawei Li, Harrison W. Smith, Craig A. Smibert, Alexander F. Palazzo

**Affiliations:** 1 School of Basic Medical Sciences, Fujian Medical University, Fuzhou, Fujian, China; 2 Department of Biochemistry, University of Toronto, Toronto, Ontario, Canada; John Hopkins University, UNITED STATES

## Abstract

Mutations in RanBP2 (also known as Nup358), one of the main components of the cytoplasmic filaments of the nuclear pore complex, contribute to the overproduction of acute necrotizing encephalopathy (ANE1)-associated cytokines. Here we report that RanBP2 represses the translation of the *interleukin 6* (*IL6*) mRNA, which encodes a cytokine that is aberrantly up-regulated in ANE1. Our data indicates that soon after its production, the *IL6* messenger ribonucleoprotein (mRNP) recruits Argonautes bound to *let-7* microRNA. After this mRNP is exported to the cytosol, RanBP2 sumoylates mRNP-associated Argonautes, thereby stabilizing them and enforcing mRNA silencing. Collectively, these results support a model whereby RanBP2 promotes an mRNP remodelling event that is critical for the miRNA-mediated suppression of clinically relevant mRNAs, such as *IL6*.

## Author summary

RanBP2 (also known as Nup358) is one of the main components of the cytoplasmic filaments of the nuclear pore complex. It has been speculated that RanBP2, which has a SUMO E3-ligase domain, may alter the composition of messenger ribonucleoprotein (mRNP) complexes as they emerge from the nuclear pore and thus regulate the ultimate fate of the mRNAs in the cytoplasm. Five separate missense mutations in RanBP2 cause acute necrotizing encephalopathy 1 (ANE1), which manifests as a sharp rise in cytokine production after common viral infections such as influenza and parainfluenza. However, how RanBP2 and its ANE1-associated mutations affect cytokine production is not well understood. Here we find that Argonaute proteins can be loaded onto mRNAs in the nucleus and upon their nuclear export become sumoylated by RanBP2. This in turn leads to the stabilization of Argonaute-mRNA complexes in the cytosol and enforces silencing of clinically relevant mRNAs, such as *interleukin-6* (*IL6*). These finding presents one of the few biochemical characterizations of an mRNP remodeling event that occurs on the nuclear pore, and opens the possibility that cells control ANE1-associated cytokines via the interplay between RanBP2, microRNA-mediated silencing pathways, and post-translational regulation of proteins.

## Introduction

Ran-binding protein 2 (RanBP2), also known as Nucleoporin 358 KDa (Nup358), is one of the main components of the cytoplasmic filaments of the nuclear pore complex [1]. It has a SUMO E3-ligase domain that post-translationally modifies several proteins [2] and has been implicated in regulating mRNA metabolism [3–5]. In particular, mRNAs are known to be packaged into messenger ribonucleoprotein (mRNP) complexes which are thought to undergo maturation events where their proteins are exchanged or post-translationally modified. Virtually all mRNPs must cross the nuclear pore prior to their translation, permitting nuclear pore filament proteins to survey the entire transcriptome and modulate its output. It has been speculated that some nuclear pore proteins, especially those present on the nucleoplasmic and cytoplasmic faces of the pore, regulate mRNP maturation events [6–11], however this is poorly understood.

Previously, we found that RanBP2 was required for the efficient translation of mRNAs that contain signal sequence coding regions (SSCRs), which code for short hydrophobic polypeptides and are found at the 5′ end of the open reading frame (ORF) of most secretory and membrane-bound proteins [5]. The majority of SSCRs in vertebrates are depleted of adenines, are enriched in GC-motifs and are present in the first exon [7,12–14]. Importantly, human RanBP2 contains eight zinc fingers that directly bind to adenine-depleted SSCRs [5]. Moreover, the ability of RanBP2 to promote translation is dependent on its zinc fingers and the presence of an adenine-depleted SSCR [5]. Overall our results suggest that upon the completion of nuclear export, mRNAs that contain adenine-depleted SSCRs directly interact with RanBP2 through its zinc fingers, and that this interaction likely modifies proteins that are associated with the mRNP in order to potentiate the translation of these mRNAs [5,7,10].

Mutations in RanBP2 have also been associated with pathology. In particular, five separate missense mutations in the N-terminal or zinc finger region of RanBP2 (T585M, T653I, I656V, T681C, and P1750R) are genetic risk factors for a pediatric neurological disease called acute necrotizing encephalopathy (ANE1) [15–17]. 40% of individuals with one of these dominant mutations secrete excessive amounts of cytokines (known as a “cytokine storm”) in response to influenza infection [18–20]. Generally, the massive secretion of cytokines include pro-inflammatory cytokines such as IL6, TNFα, IL10, IFNγ, sTNFα receptor, and IL15 [21–27] (S1 Table). The resulting elevated levels of cytokines infiltrate into the cerebral spinal fluid, causing neuropathology, seizures, coma and a high rate of mortality. Those who survive often suffer from long-term neurological damage. However, how mutations in RanBP2 contribute to the overproduction of ANE1-associated cytokines remains unclear.

The cytokine that has been best documented to be upregulated during ANE1 is interleukin 6 (IL6) (S1 Table). The expression of this cytokine has been the subject of much investigation. One of the key ways in which IL6 is regulated is by the *let-7* microRNA (miRNA), which recognizes one binding site in the 3′ untranslated region (UTR) of the *IL6* mRNA [28,29]. Indeed, many infections are known to modulate the expression of *let-7* miRNA family members and *let-7* in turn modulates the inflammatory response [28–34]. miRNAs, such as *let-7*, associate with the RNA Induced Silencing Complex (RISC) to silence their targets, and the main component of this complex, the Argonaute (AGO) proteins, are regulated by post-translational modifications, such as ubiquitination and sumoylation, which in turn affect their activity and stability [35–40]. Interestingly, a recent report indicates that RanBP2 is required for *let-7*-mediated gene silencing [41]. These observations suggest that RanBP2 might impact the translation of the *IL6* mRNA by post-translational regulation of AGO proteins.

Here we present evidence that RanBP2 promotes the *let-7*-mediated suppression of IL6 protein production by sumoylating AGO1, which antagonizes AGO1 ubiquitination and thus promotes its stability and its ability to translationally silence the *IL6* mRNA. Furthermore, we observe that Argonaute proteins associate with *IL6* mRNA in the nucleus, and then likely accompany the mRNA through the pore. Our data suggests that when this mRNP reaches the cytoplasm, RanBP2 sumoylates AGO1, and likely AGO2, thereby stabilizing the Argonaute-mRNA complex and promoting *IL6* mRNA silencing. Thus, our work provides one of the few examples of how mRNPs are subjected to a maturation event at the pore, and how these maturation events affect the ultimate fate of the mRNAs in the cytoplasm.

## Results

### RanBP2 inhibits the translation of an *IL6-HA* reporter mRNA

**Fig 1:**
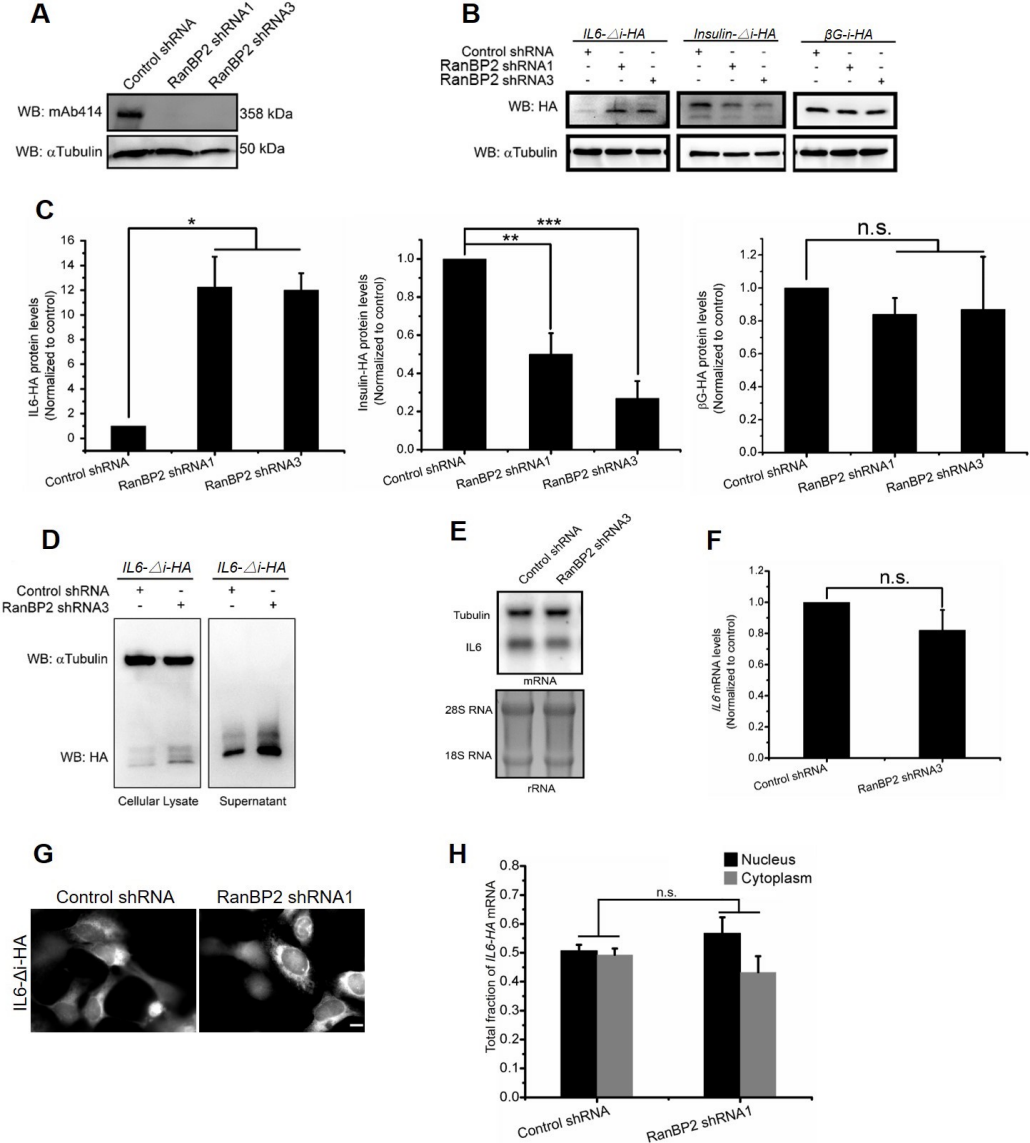
RanBP2 suppresses the translation of *IL6* mRNA. (A) U2OS cells were infected with lentivirus containing shRNA1 or shRNA3 directed against RanBP2, or scrambled shRNA (“control shRNA”). Four days post-infection, cell lysates were collected, separated by SDS-PAGE, and immunoblotted for nucleoporins using mAb414, which recognizes RanBP2, and α-tubulin as a loading control. (B-C) U2OS cells were infected with lentivirus that delivered shRNA1 or shRNA3 against RanBP2 or control virus. Three days post-infection, cells were transfected with plasmids containing either the *IL6-Δi-HA*, *insulin-Δi-HA*, or *β-globin-i-HA* genes. 18–24 h post-transfection cell lysates were collected and separated by SDS-PAGE. The level of each protein was analyzed by immunoblot for HA, and α-tubulin as a loading control (B). The levels of each HA-tagged protein and α-tubulin were quantified using densitometry analysis. The HA/tubulin ratio was normalized to control shRNA-treated cells and plotted, with each bar representing the average of three independent experiments ± SEM (C). (D) As in (B) except that cell lysates (left panel) or supernatant precipitated by TCA (right panel) were collected, separated by SDS-PAGE and immunoblotted with antibodies against HA and α-tubulin. (E-F) As in (B) except that RNA was purified from cell lysates and separated on a denaturing agarose gel. The levels of *IL6-HA* mRNA and *α-tubulin* were assessed by northern blot, while the ribosomal RNA was detected by ethidium bromide (E). *IL6-HA* and *α-tubulin* mRNA levels were quantified using densitometry analysis. The *IL6-HA*/*tubulin* ratio was normalized to control shRNA-treated cells and plotted with each bar representing the average of three independent experiments ± SEM (F). (G-H) Control and RanBP2-depleted cells were transfected with an intronless version of *IL6-HA* (*IL6-Δi-HA*) plasmid for 14–18 hr, then fixed, permeabilized, and stained for mRNA using a fluorescent in situ hybridization (FISH) probe directed against *IL6*. The cells were imaged (G) and total integrated fluorescence was assessed in the cytoplasm and nucleus (H). For each experiment at least 20 cells were assessed with each bar representing the average of three independent experiments ± SEM. Scale bar = 10 μm. *P = 0.01–0.05, **P = 0.001–0.01, ***P < 0.001, n.s. indicates no significant difference (Student’s *t*-test).

We had previously found that RanBP2 is required for the efficient translation of mRNAs that contain signal sequence coding regions (SSCRs) and hence encode secretory proteins. However, it appeared that mRNAs that encode ANE1-associated cytokines, which are also secreted, had SSCRs that lacked features associated with RanBP2-depedent upregulation (see S1 Text and S1 Fig). In light of this, we determined whether RanBP2 regulated the expression of ANE1-associated cytokines. Of all the cytokines overproduced in ANE1-patients, IL6 has been the best documented (S1 Table). We thus depleted RanBP2 using lentiviral delivered shRNAs (Fig 1A) and examined the expression of a C-terminally tagged IL6 expressed off of a transfected plasmid. This reporter included the IL6 5′ and 3′ UTRs, which are known to be important for its regulation [42–46]. Unexpectedly, we found that RanBP2-depletion resulted in a ~12 fold increase in intracellular IL6-HA when compared to control cells (Fig 1B and 1C). This increase was also seen for secreted IL6-HA (Fig 1D). This was in stark contrast to the expression of the *insulin-HA* reporter, whose SSCR has features associated with RanBP2-dependent upregulation, and whose translation was reduced in RanBP2-depeleted cells (Fig 1B and 1C), a result that was consistent with our previous findings [5]. Protein production from the *β-globin-HA* mRNA, which lacks an SSCR, was unaffected by RanBP2-depletion (Fig 1B and 1C).

The increase in IL6-HA protein synthesis was not due to changes in the total level of *IL6* mRNA (Fig 1E and 1F), or to changes in the distribution of *IL6* mRNA between the cytoplasm and the nucleus (Fig 1G and 1H). Indeed, we and other have documented that RanBP2-depletion had no detectable effects on mRNA nuclear export in human cells [5,47]. Previous studies have demonstrated that splicing can potentiate the efficiency of translation [48], and the main *IL6* isoform has four introns. However, versions of the *IL6* reporter that either contained the endogenous first intron (*IL6-1i*), or the intron of *fushitarazu* mRNA (*ftz*), at the first intron site (*IL6-1f*), still produced more protein after RanBP2-depletion, indicating that this effect is independent of splicing (S2 Fig).

From these results, and from the results of our polysome profiling (see below), we conclude that RanBP2 inhibits IL6-HA protein production from a transfected reporter construct. As RanBP2-depletion did not affect the levels or the cytoplasmic/nuclear distribution of the reporter mRNA, we concluded that RanBP2 inhibits the translation of *IL6-HA* mRNA.

### The SUMO E3-ligase domain of RanBP2 is required for the repression of IL6

**Fig 2:**
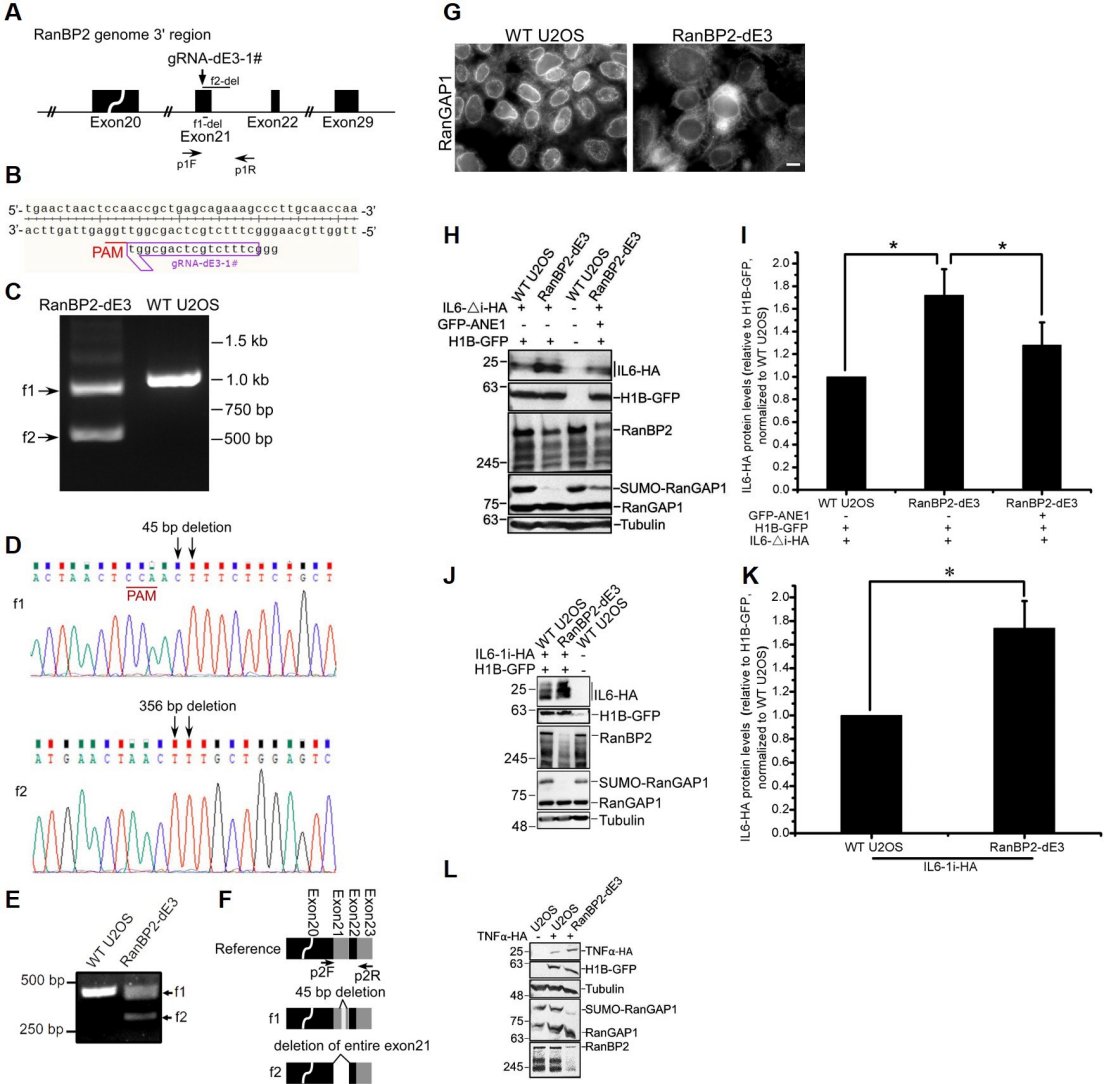
The SUMO E3-ligase domain of RanBP2 is required for the repression of IL6 in U2OS cells. The SUMO E3-ligase domain of RanBP2 was targeted by CRISPR/Cas9 in U2OS cells. (A) A schematic diagram of the region of the RanBP2 gene targeted by CRISPR/Cas9 loaded with the guide RNA, “gRNA-dE3-1#”, whose sequence is shown in (B). Also indicated are the PCR amplification primers “p1F” and “p1R”, and the regions deleted “f1-del” and “f2-del” in each *RanBP2* allele present in the cell clone “RanBP2-dE3”. (C) PCR amplification, using p1F and p1R primers, of the genomic region targeted by gRNA-dE3-1#. Note that the reaction from the RanBP2-dE3 cell clone lysates produced two amplicons (“f1” and “f2”), which were both smaller than the amplicon produced from unmodified wildtype U2OS cells (“WT U2OS”). (D) Sequencing of the two PCR products (f1 and f2) in (C). Note that the length of the deletion in each *RanBP2* gene allele are indicated. (E) cDNA amplification, using p2F and p2R primers indicated in (F), of *RanBP2* mRNA. Note that the reactions with RanBP2-dE3 cell lysates produced two amplicons, corresponding to the two mRNA forms from f1 and f2 gene copies in (C-D). (F) Schematic of the modified regions in the *RanBP2* mRNAs. Also indicated are the PCR primers “p2F”and “p2R” for cDNA amplification, and the regions deleted in mRNAs produced from each *RanBP2* allele in RanBP2-dE3 cells. (G) Unmodified and RanBP2-dE3 U2OS cells were fixed, immunostained using anti-RanGAP1 antibody and imaged by epifluorescence microscopy. Scale bar = 10 μm. (H-I) Various U2OS cell lines (unmodified, RanBP2-dE3 and RanBP2-dE3 that stably express GFP-tagged RanBP2 which contains 3 ANE1-associated mutations, “GFP-ANE1”) were transfected with plasmids containing an intronless version of *IL6-HA* (*IL6-Δi-HA*) and *histone 1B-GFP* (*H1B-GFP*). Cell lysates were collected 24 h post-transfection and separated by SDS-PAGE. Proteins were detected with by immunoblot with antibodies against HA, GFP, RanBP2, RanGAP1 and α-tubulin. Note that H1B-GFP was used as a control for transfection and general mRNA translation while α-tubulin was used as a loading control. Also note that RanBP2-dE3 cells had lower expression of RanBP2 and lacked sumoylated-RanGAP1. This sumoylation pattern was re-established by the expression of GFP-ANE1. The level of GFP-ANE1 was less than the level of RanBP2 present in unmodified U2OS cells (H). IL6-HA and H1B-GFP protein levels were quantified using densitometry analysis and the ratio of IL6-HA/H1B-GFP was normalized to unmodified U2OS cells, with each bar representing the average of three independent experiments ± SEM (I). (J-K) Same as (H-I), except that an intron-containing *IL6-HA* construct (*IL6-1i-HA*) was used. (L) As in (H), except that TNF-α-HA was transfected. *P = 0.01–0.05 (Student’s *t*-test).

Next, we investigated whether the SUMO E3-ligase activity of RanBP2 is required to inhibit *IL6* mRNA translation. We used CRISPR/Cas9 with a specific guide RNA (“gRNA-dE3-1#”) to target the E3 domain of RanBP2 in U2OS cells (Fig 2A and 2B). We obtained a clone, called RanBP2 dead E3 (hereafter referred to as RanBP2-dE3), where one copy of the gene (“f1”; Fig 2A–2D) had a 45 base pair (bp) deletion just downstream from the targeted region (i.e., the guide RNA Protospacer Adjacent Motif “PAM” site) that eliminated 15 amino acids in the SUMO E3-ligase domain, and where the second copy (“f2”; Fig 2A–2D) had a 356 bp deletion which eliminated the remaining part of exon 21 and a portion of the following intronic sequence (Fig 2A). When cDNA was sequenced from this cell line, we could detect two mRNA forms, one that corresponds to the 45 bp deletion, as is expected from the f1 gene copy, and a second mRNA where exon 21 is skipped, which presumably came from the f2 gene copy (Fig 2E and 2F). Since exon 21 has 171 nucleotides, its elimination does not alter the reading frame of downstream exons. Both altered proteins are expected to disrupt RanBP2’s E3 domain (S3A and S3B Fig), which binds to Ubc9, the only known SUMO-conjugating E2 enzyme in humans, and SUMO-conjugated RanGAP1 (SUMO-RanGAP1) [49]. In agreement with this, the nuclear rim localization of RanGAP1 was disrupted in RanBP2-dE3 cells (Fig 2G) despite the fact that the mutant RanBP2 protein(s) was still at the nuclear rim (S4A Fig). RanBP2-dE3 cells also had significantly decreased levels of RanBP2 protein, although this appeared to vary greatly between experiments (for example compare levels of RanBP2 in RanBP2-dE3 cells in Fig 2H and 2J), and had almost no detectable SUMO-RanGAP1 (Fig 2H and 2J). Importantly, when RanBP2-dE3 cells were transfected with *IL6-△i-HA* or *IL6-1i-HA*, these cells had significantly elevated IL6-HA protein expression over control unmodified wildtype (“WT”) cells (Fig 2H–2K). Note that co-transfected Histone1-GFP (“H1B-GFP”) expression was similar in both cell lines (Fig 2H and 2J), indicating that there wasn’t a general alteration in mRNA translation. We also examined the expression of TNFα, another ANE1-associated cytokine (S1 Table). As with IL6, the level of TNFα-HA protein increased in RanBP2-dE3 cells (Fig 2L).

**Fig 3:**
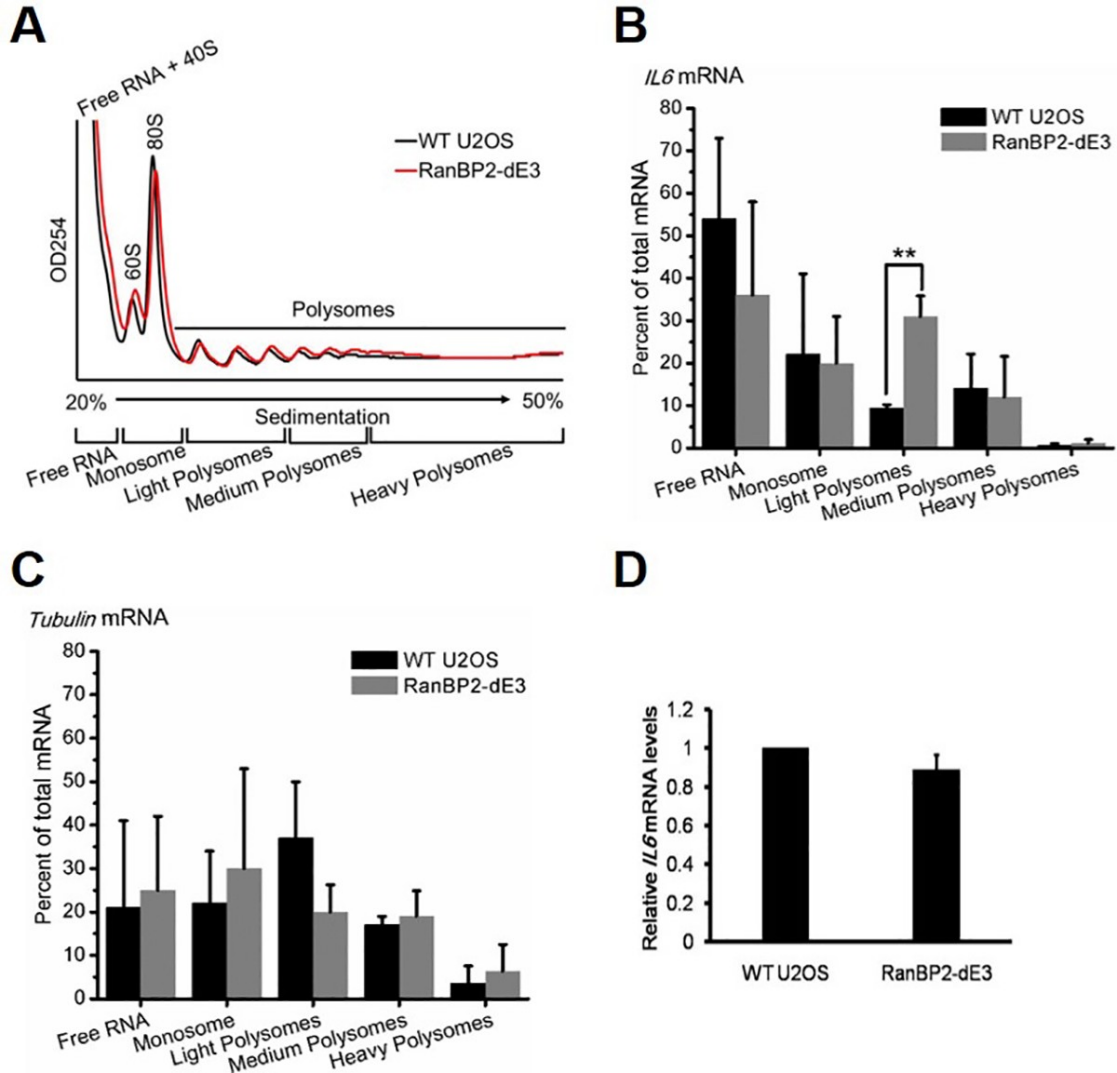
The SUMO E3-ligase domain of RanBP2 is required for the translational repression of *IL6* mRNA. Unmodified and RanBP2-dE3 U2OS cells were transfected with an intron-containing *IL6-HA* construct (*IL6-1i-HA*). 24 h post-transfection, cells were lysed and fractionated by centrifugation over a sucrose gradient (A-C) or directly analyzed by RT-qPCR (D). (A) OD254 trace of the sucrose gradients to determine the distribution of monosomes and polysomes. RT-qPCR of *IL6* (B) and *α-tubulin* (C) mRNA were normalized against *luciferase* mRNA that was spiked into each fraction to control for RNA recovery. Note the significant increase in *IL6* mRNA in the polysome fraction in RanBP2-dE3 cells. (D) *IL6-HA* and *α-tubulin* mRNA levels were measured by RT-qPCR. The *IL6-HA*/*tubulin* ratio was normalized to unmodified U2OS cells and each bar representing the average of three independent experiments ± SEM. **P = 0.001–0.01 (Student’s *t*-test).

To confirm that the effect on IL6 expression was due to a decrease in translation, we fractionated lysates from unmodified and RanBP2-dE3 U2OS cells on a sucrose gradient (Fig 3A) and assessed the distribution of mRNA by RT-qPCR (Fig 3B and 3C). We found that *IL6-HA* mRNA was shifted towards the polysome fraction in mutant cells (Fig 3B). Meanwhile, the distribution of *α-tubulin* mRNA remained relatively unaffected (Fig 3C). Total *IL6-HA* mRNA levels also remained unaffected (Fig 3D). These data indicate that either the SUMO E3-ligase activity, or high levels of RanBP2, are required for the translational repression of the *IL6* mRNA.

**Fig 4:**
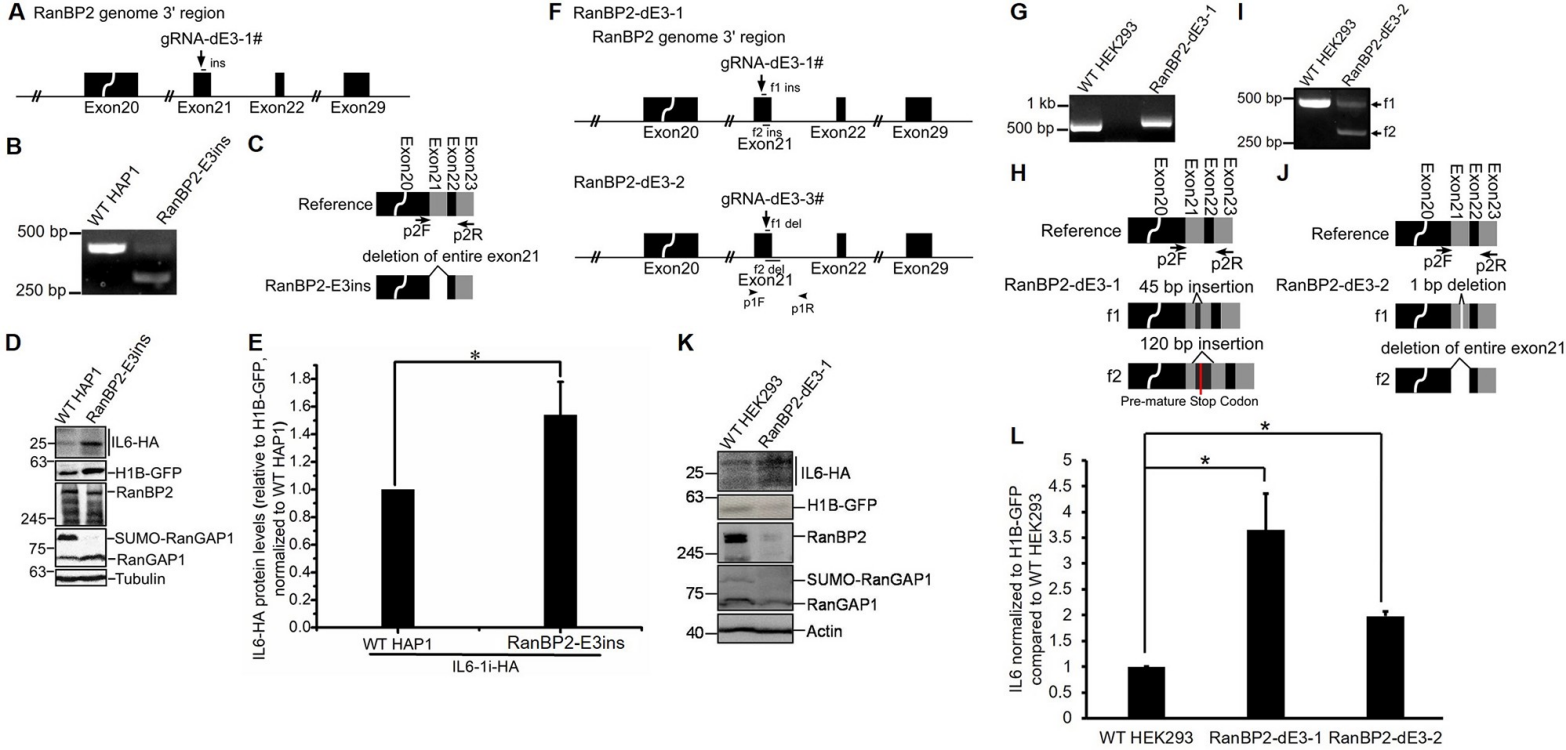
The SUMO E3-ligase domain of RanBP2 is required for the repression of IL6 in HAP1 and HEK293 cells. The SUMO E3-ligase domain of RanBP2 was targeted by CRISPR/Cas9 in HAP1 cells (A-E) and HEK293 cells (F-L). (A) A schematic diagram of the region of the RanBP2 gene in HAP1 cells targeted by CRISPR/Cas9 loaded with the guide RNA “gRNA-dE3-1#”. Note that the insertion “ins” in the RanBP2-E3ins cell line is indicated. (B) cDNA amplification, using p2F and p2R primers indicated in (C), of *RanBP2* mRNA. Note that the reaction with the RanBP2-E3ins cell lysates produced an amplicon which was smaller than from unmodified HAP1 cells (“WT HAP1”). (C) Schematic of the modified region in the *RanBP2* mRNA. Also indicated are the PCR primers “p2F”and “p2R” for cDNA amplification. Note that the mRNA produced from the modified RanBP2-E3ins gene lacks exon 21. (D-E) Unmodified and RanBP2-E3ins HAP1 cells were transfected with an intron-containing version of *IL6-HA* (*IL6-1i-HA*) and *histone 1B-GFP* (*H1B-GFP*). Cell lysates were collected 24 h post-transfection, and IL6-HA was immunoprecipitated with mouse anti-HA antibody and protein G beads (Sigma), separated by SDS-PAGE and immunoblotted with a rabbit anti-HA antibody (top panel). For the detection of other proteins, cell lysates were directly separated by SDS-PAGE and immunoblotted with antibodies against GFP, RanBP2, RanGAP1 and α-tubulin. Note that RanBP2-E3ins cells lacked sumoylated RanGAP1 but expressed RanBP2 at similar levels to unmodified HAP cells (D). IL6-HA and H1B-GFP protein levels were quantified using densitometry analysis and the ratio of IL6-HA/H1B-GFP was normalized to unmodified HAP1 cells, with each bar representing the average of three independent experiments ± SEM (E). (F) A schematic diagram of the region of the RanBP2 gene in HEK293 cells targeted by CRISPR/Cas9 loaded with the guide RNA “gRNA-dE3-1#” to generate the RanBP2-dE3-1 clone or with “gRNA-dE3-3#” to generate the RanBP2-dE3-2 clone. Also indicated are the PCR amplification primers “p1F” and “p1R”, and the regions inserted “f1-ins” and “f2-ins” or regions deleted “f1-del” and “f2-del” in each *RanBP2* allele present in the “RanBP2-dE3” clones. (G-I) cDNA analysis similar to (B-C), except for RanBP2-dE3-1 in (G-H) and for RanBP2-dE3-2 in (I-J). (K-L) As in (D-E), except that unmodified, RanBP2-dE3-1 and RanBP2-dE3-2 HEK293 cells were transfected, and cell lysates were directly analyzed by SDS-PAGE and immunoblotting. Note that RanBP2-dE3-1 and RanBP2-dE3-2 HEK293 cells were not assessed in parallel, but each compared to their own parental unmodified cells. The ratio of IL6-HA/HIB-GFP in each RanBP2-dE3 cell line were normalized to unmodified parental HEK293 cells, with each bar representing the average of two independent experiments ± SEM. *P = 0.01–0.05 (Student’s *t*-test).

To validate our findings, we also generated RanBP2 sumoylation-deficient mutants in human HAP1 and HEK293 cells. As HAP1 are haploid, we only had to modify the single copy of the *RanBP2* gene. Using a similar strategy as described above for RanBP2-dE3 generation, we isolated a mutant HAP1 clone called RanBP2-E3 insertion mutant (hereafter referred to as RanBP2-E3ins), which had a 51 bp insertion into the middle of the E3 domain (Figs 4A and S5A and S5B). When this region was amplified from total cDNA and sequenced, we found that the mutation led to the skipping of exon 21 (Fig 4B and 4C), which is predicted to disrupt the E3 domain as was the case in the U2OS mutant cells (S3C Fig). Although RanBP2-E3ins had no effect on RanBP2 protein level, it eliminated RanGAP1 sumoylation (Fig 4D) and upregulated protein production from the *IL6-1i-HA* reporter when normalized to H1B-GFP expression (Fig 4D and 4E).

We then took the same approach and modified HEK293 cells. We isolated two mutant cell lines (RanBP2-dE3-1 and RanBP2-dE3-2; Fig 4F). The first mutant cell line had insertions into its two copies of RanBP2 (f1 ins had a 45 bp insertion, and f2 had a 120 bp insertion that contained a pre-mature stop codon, S5C and S5D Fig), both of which were incorporated into its mRNA, as detected by cDNA amplification and sequencing (Figs 4G and 4H and S3D). It should be noted that only 1 of the 10 sequenced cDNA clones had the 120 bp insertion, suggesting that this mRNA was subjected to non-sense mediated decay. The second mutant HEK293 cell line had versions of the RanBP2 gene with a 1 bp deletion (f1) and with a 123 bp deletion (f2) (S5E and S5F Fig). From the cDNA amplification and sequencing we could detect *RanBP2* mRNA with the 1 bp deletion from copy f1 (despite the fact that it should trigger nonsense-mediated decay) and mRNA missing exon 21, which again we presume was made from the f2 copy of the gene (Figs 4I and 4J and S3E). As is the case with the mutant U2OS and HAP1 cell lines, both mutant HEK293 cell lines had elevated levels of IL6-HA protein and a drastic reduction in RanGAP1 sumoylation (Fig 4K and 4L).

Taken together, these results demonstrate that either the SUMO-E3 domain of RanBP2, or high levels of RanBP2, is required to repress IL6-HA reporter protein production in various human cell lines.

### RanBP2 with ANE1-associated mutations rescues the translational repression of the *IL6-HA* reporter mRNA

To evaluate the effect of the ANE1 mutant on the suppression of IL6 production, we isolated a clone of RanBP2-dE3 U2OS cells that stably expressed an N-terminal GFP-tagged RanBP2 bearing 3 of the ANE1 mutations (T585M, T653I, and I656V). This mutant localized to the nuclear rim (S4B Fig) and rescued IL6-HA repression in RanBP2-dE3 cells (Fig 2H and 2I), although it only partially restored SUMO-RanGAP1 (Fig 2H). It should be noted that the expression level of this protein was low (Fig 2H), suggesting that low levels of RanBP2, and of its associated SUMO E3-ligase activity, is sufficient for RanBP2 to suppress IL6 expression.

From these results we conclude that since low levels of the triple ANE1 mutant rescued the suppression of IL6-HA, it is unlikely that cells with mutations in the E3 domain are unable to repress IL6 due to low levels of RanBP2. Furthermore, our results indicate that the primary defect of the ANE1 mutations is not the general misregulation of IL6 expression. However, it remains possible that the ANE1 mutations may impact the expression of IL6 and/or other critical proteins only in the relevant cell line, or in viral-infected cells.

### The *let-7* binding site within the *IL6* 3′UTR is required for RanBP2-suppression of IL6 protein production

**Fig 5:**
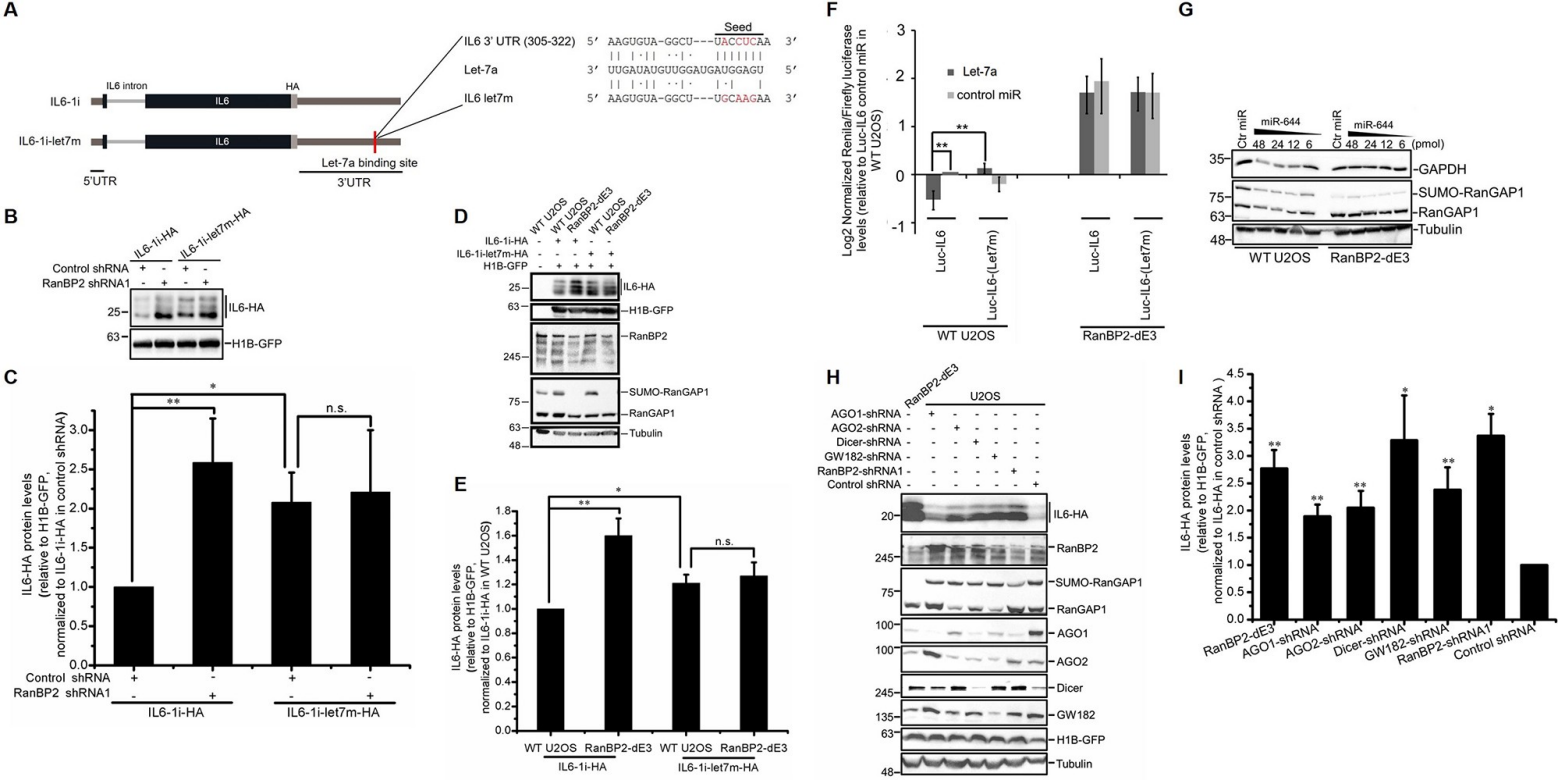
RanBP2 silences *IL6* mRNA through its *Let7*-binding site. (A) Schematic of the intron-containing *IL6-HA* construct (*IL6-1i*), without or with a mutation that eliminated the *let-7* miRNA binding site (*IL6-1i-let7m*). (B-C) U2OS cells treated with lentivirus that delivered shRNA1 against RanBP2, or scrambled shRNA (“control shRNA”) were co-transfected with the *IL6-1i* constructs in (A) and *histone 1B-GFP* (*H1B-GFP*). 24 h post-transfection, cell lysates were collected and separated by SDS-PAGE. The level of IL6-HA and GFP were analyzed by immunoblot (B). The IL6-HA/H1B-GFP ratio was quantified using densitometry analysis, normalized to *IL6-1i-HA*-transfected control shRNA-treated cells, and plotted (C). (D-E) Unmodified and RanBP2-dE3 U2OS cells were transfected with *IL6-1i-HA* or *IL6-1i-Let7m-HA* and *H1B-GFP*. Cell lysates were collected 24 h post-transfection and separated by SDS-PAGE. Proteins were detected with by immunoblot with antibodies against HA, GFP, RanBP2, RanGAP1 and α-tubulin (D). IL6-HA and H1B-GFP protein levels were quantified using densitometry analysis and the ratio of IL6-HA/H1B-GFP was normalized to *IL6-1i-HA* transfected unmodified U2OS cells (E). (F) Unmodified and RanBP2-dE3 U2OS cells were co-transfected with *Let7a* miRNA or a scrambled control miRNA (“control miR”), and a dual luciferase plasmid that contains a *Renilla luciferase* reporter plasmid, carrying either the wild-type 3′UTR of *IL6* (*Luc-IL6*) or the *Let7a* binding site mutant (*Luc-IL6-(Let7m*), see Fig 5A for the sequence), and the Firefly *luciferase* as an internal control. 24 h after transfection, Renilla and Firefly luciferase luminescence were measured, and the ratio was normalized to unmodified U2OS cells transfected with *Luc-IL6* and control miR. (G) Unmodified and RanBP2-dE3 U2OS cells were transfected with various amounts of miR-644 or 48 pmol of miR-144 (“Ctr miR”). 24 h post-transfection, cell lysates were collected, separated by SDS-PAGE, and immunoblotted for GAPDH, RanGAP1 and α-tubulin. (H-I) Unmodified U2OS cells were treated with lentivirus that delivered shRNAs against various proteins, or scrambled shRNA (“control shRNA”), and were transfected with plasmid containing the *IL6-1i-HA* construct. For comparison, RanBP2-dE3 U2OS cells were also included in this analysis. Cell lysates were collected 24 h post-transfection and separated by SDS-PAGE. Proteins were detected by immunoblot with antibodies against HA, RanBP2, RanGAP1, AGO1, AGO2, Dicer, GW182, GFP and α-tubulin (H). IL6-HA and H1B-GFP protein levels were quantified using densitometry analysis and the ratio of IL6-HA/H1B-GFP was normalized to *IL6-1i-HA* transfected unmodified U2OS cells (I). Each bar is the average of three independent experiments ± SEM. *P = 0.01–0.05, **P = 0.001–0.01, n.s. indicates no significant difference (Student’s *t*-test).

By analyzing IL6-HA deletion and chimeric constructs, we determined that regions in the 5′ and 3′UTRs were both required for RanBP2-dependent regulation (S1 Text and S6 Fig). We noted that the RanBP2-responsive region in the *IL6* 3′UTR contained a *let-7a* miRNA binding site. Previously it was reported that *let-7a* miRNA directly inhibits expression of IL6 through this site [28,29], and that RanBP2 is required for *let-7a*-mediated translational suppression [41]. To determine whether the *let-7* binding site is required for RanBP2-mediated suppression of IL6, we generated a mutant of the *IL6-1i-HA* reporter bearing 4 point mutations in the *let-7* recognition site (*IL6-1i-Let7m-HA*) (Fig 5A). As expected, the *IL6-1i-Let7m-HA* construct produced more protein compared to the *IL6-1i-HA* construct in control U2OS cells (Fig 5B and 5C). In contrast, the level of protein from *IL6-1i-Let7m-HA* was similar to *IL6-1i-HA* in RanBP2-depleted cells (Fig 5B and 5C). Similar results were obtained in RanBP2-dE3 U2OS cells (Fig 5D and 5E). Note that the level of protein generated from co-transfected H1B-GFP was similar in all the cell lines (Fig 5B and 5D).

To further confirm these results, we monitored the expression of Renilla *luciferase* reporter plasmids carrying the wild-type 3′UTR of *IL6* (*Luc-IL6*) or a mutant version that lacked the *let-7* binding site (*Luc-IL6-(Let7m)*) [29] in cells co-transfected with miRNA mimics. These reporter plasmids also contained the Firefly *luciferase* gene, to control for general changes in gene expression. In control U2OS cells we observed that co-transfection of *let-7a* inhibited protein production from the *Luc-IL6* reporter, but did not impact expression from *Luc-IL6-(Let7m)* (Fig 5F), as reported by others [29]. Importantly, RanBP2-dE3 U2OS cells had significantly enhanced expression from the *Luc-IL6* construct, in comparison to the unmodified cells (Fig 5F). Moreover, in the RanBP2-dE3 cells introduction of *let-7a* did not repress *Luc-IL6* (Fig 5F).

These results indicate that RanBP2 represses IL6 expression in part through *let-7*-mediated translational suppression of the *IL6* mRNA. The fact that the RanBP2-dE3 cells have an overall higher expression of all the *Luc-IL6* constructs, regardless of the presence of a *let-7* binding site, suggests that there may be other RanBP2-sensitive elements in the 3′UTR (e.g. other miRNA binding sites).

### RanBP-dE3 cells have a general miRNA-silencing defect

To determine whether the defect in *let-7*-mediated silencing extends to other miRNAs, we transfected unmodified and RanBP2-dE3 U2OS cells with miR-644, which is known to repress the expression of GAPDH [50]. In agreement with the idea that miRNA silencing is generally compromised in RanBP2-dE3 cells, the presence of miR-644 did not lead to a decrease in GAPDH levels in these cells, as it did in the wildtype U2OS cells (Fig 5G).

### RISC is required to silence IL6

Our data and previous results indicate that IL6 is regulated by the RISC complex. To confirm this, we depleted various proteins in the RNA interference (RNAi) pathway and monitored IL6-HA production. Depletion of AGO1, AGO2, Dicer, and GW182 all led to an increase in IL6-HA protein production without affecting co-transfected H1B-GFP (Fig 5H and 5I). Interestingly, depletion of certain factors in this pathway led to changes in the levels of other factors, suggesting that these proteins regulate each other. For example, the depletion of every RISC-associated protein, as well as RanBP2, led to a precipitous drop in AGO1 levels (Fig 5H). The level of AGO1 was also low in RanBP2-dE3 cells in comparison to unmodified U2OS cells. In contrast, depletion of AGO1 led to the upregulation of AGO2 protein levels (Fig 5H). To rule out that changes in AGO1 were due to IL6-HA production, we repeated these experiments in untransfected cells. Again, RanBP2-depletion or elimination of RanBP2-dependent sumoylation led to a drop in AGO1 levels (S7 Fig). Furthermore, expression of the ANE1 mutant RanBP2 in RanBP2-dE3 cells restored AGO1 levels (S7B Fig), correlating with the fact that silencing of *IL6* mRNA is restored in this cell line (Fig 2H and 2I). In all these experiments AGO2 levels remained relatively unaffected (S7 Fig).

In summary, our data suggested that not only was IL6 expression inhibited by RISC, but that RanBP2 may regulate IL6 by affecting AGO1 levels in U2OS cells.

### RanBP2 regulates IL6 translation by stabilizing AGO1 in U2OS cells

**Fig 6:**
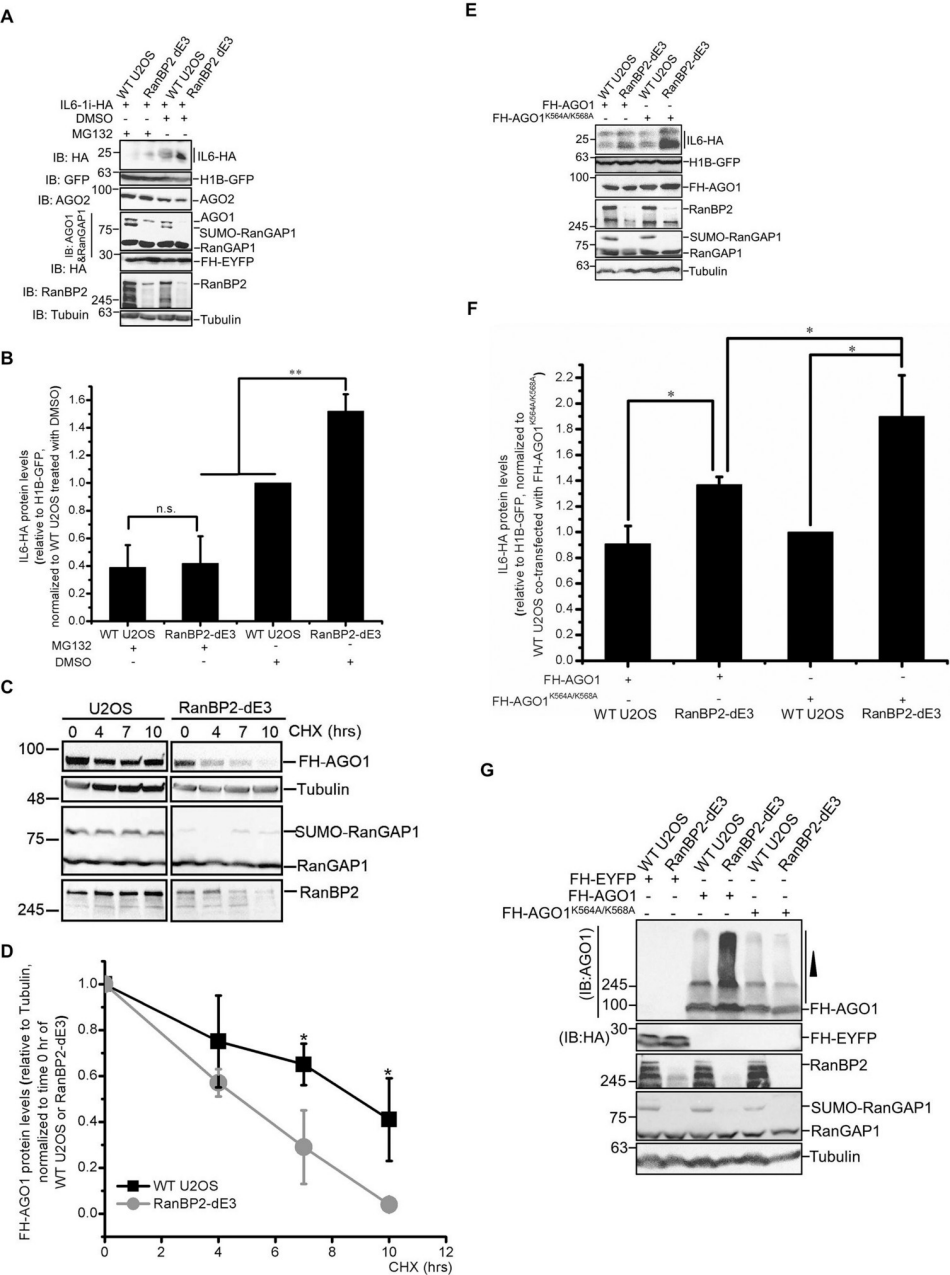
RanBP2 represses IL6 expression by promoting AGO1 stabilization. (A-B) Unmodified and RanBP2-dE3 U2OS cells were co-transfected with plasmids for an intron-containing IL6 construct (*IL6-1i-HA*), histone 1B-GFP (*H1B-GFP*) and Flag-HA-tagged yellow fluorescent protein (*FH-EYFP*). 18 h post-transfection, cells were treated with MG132 (10 μM) (MG132 “+”), or DMSO (MG132 “-”), for an additional 7 h. Cell lysates were collected, separated by SDS-PAGE, and immunoblotted with antibodies against HA, GFP, AGO2, AGO1, RanGAP1, RanBP2 and α-tubulin (A). Note that AGO1 and RanGAP1 were blotted together. Also note that DMSO-treated RanBP2-dE3 cells had no detectable AGO1. MG132-treatment led to an increase in AGO1, AGO2 and mutant RanBP2-dE3 levels. In contrast, the same treatment led to a decrease in IL6-HA levels. IL6-HA and H1B-GFP protein levels were quantified using densitometry analysis and the ratio of IL6-HA/H1B-GFP was normalized to DMSO-treated unmodified U2OS cells (B). Each bar is the average of three independent experiments ± SEM. (C-D) Unmodified and RanBP2-dE3 U2OS cells were transfected with Flag-HA-tagged AGO1 (*FH-AGO1*). 24 h post-transfection, the cells were treated with cycloheximide (CHX, 100 μM) for various amounts of time to block further translation and thus allowing us to determine the rate of AGO1 turnover. Cell lysates were collected, separated by SDS-PAGE, and immunoblotted with antibodies against HA (FH-AGO1), α-tubulin, RanGAP1 and RanBP2 (C). For each time point, FH-AGO1 and α-tubulin protein levels were quantified using densitometry analysis and the ratio of FH-AGO1/α-tubulin was normalized to the zero time point (D). (E-F) Unmodified and RanBP2-dE3 U2OS cells were co-transfected with *IL6-1i-HA*, *H1B-GFP* and either *FH-AGO1*, or *FH-AGO1*^*K564A/K568A*^. Cell lysates were collected 24 h post-transfection and separated by SDS-PAGE and immunoblotted with antibodies against HA (IL6-HA and FH-AGO1), GFP, RanBP2, RanGAP1 and α-tubulin (E). IL6-HA and H1B-GFP protein levels were quantified using densitometry analysis and the ratio of IL6-HA/H1B-GFP was normalized to FH-EYFP transfected unmodified U2OS cells (F). (G) Unmodified and RanBP2-dE3 U2OS cells were transfected with either *FH-AGO1*, *FH-AGO1*^*K564A/K568A*^ or *FH-EYFP*. Cell lysates were collected 24 h post-transfection, separated by SDS-PAGE and immunoblotted with antibodies against HA (FH-AGO1 and FH-EYFP), RanBP2, RanGAP1, and α-tubulin. Each bar is the average of three independent experiments ± SEM. *P = 0.01–0.05, **P = 0.001–0.01, n.s. indicates no significant difference (Student’s *t*-test).

Previously it had been reported that RanBP2 was required for the degradation of AGO2 [35], however in another report, depletion of RanBP2 had no effect on AGO2 levels [41]. Instead, this study found that RanBP2 promoted the association of miRISC with its target mRNAs [41]. Our new data suggested that RanBP2-dependent sumoylation was required for the stabilization of AGO1, but not AGO2, in U2OS cells. In agreement with this, treating RanBP2-dE3 U2OS cells with the proteasome inhibitor, MG132, increased AGO1 levels to that of unmodified cells (Fig 6A). Interestingly, MG132-treatment also increased the levels of the mutant RanBP2 in RanBP2-dE3 cells (Fig 6A), suggesting that both mutant forms are relatively unstable. This instability was not due to a lack of sumoylation activity, as depletion of Ubc9, which is required for SUMO-ligation, had no effect on RanBP2 levels (S8 Fig). MG132-treatment had no major effects on the levels of wildtype RanBP2, α-tubulin or RanGAP1 (Fig 6A). Remarkably, RanBP2-dE3 and unmodified U2OS cells treated with MG132 had much lower levels of IL6-HA than untreated cells (Fig 6A and 6B). Furthermore, MG132-treated RanBP2-dE3 and wildtype cells had similar levels of IL6-HA protein (Fig 6A and 6B), suggesting that when AGO1 degradation is inhibited, RanBP2-dependent sumoylation was no longer required for IL6 suppression in U2OS cells. Notably, this treatment did not alter global translation patterns as the levels of expression from other co-transfected reporters (H1B-GFP and Flag-HA-tagged EYFP) were similar across all cell lines and all treatments (Fig 6A).

To confirm that AGO1 stability requires RanBP2-dependent sumoylation, we treated cells with cycloheximide to prevent further expression of protein, and then assessed the rate at which the remaining AGO1 degraded. Since levels of endogenous AGO1 in RanBP2-dE3 U2OS cells were very low, we monitored the levels of overexpressed Flag-HA-tagged AGO1 (FH-AGO1). Note that the presence of an N-terminal tag is known not to interfere with Argonaute-dependent silencing [51]. Indeed, we observed that FH-AGO1 had a higher turnover rate in RanBP2-dE3 cells compared to unmodified U2OS cells (Fig 6C and 6D) and that inhibition of degradation with MG132 eliminated the difference in the levels of FH-AGO1 between these two cell lines (S9 Fig).

Finally, we tested whether the lack of silencing in RanBP2-dE3 cells can be overcome by overexpressing AGO1. Indeed, overexpression of FH-AGO1 partially suppressed the expression of IL6-HA protein in these cells when compared to the expression of mutant AGO1 (FH-AGO1^K564A/K568A^) (Fig 6E and 6F), which does not bind to miRNAs [52]. Expression of FH-AGO1 in control U2OS cells did not further reduce IL6-HA levels, suggesting that endogenous Argonautes are sufficient to suppress IL6-HA expression (Fig 6E and 6F). Expression of FH-AGO1 had no effect on H1B-GFP expression (Fig 6E) indicating that general translation was not perturbed in these cells.

From these experiments we conclude that RanBP2-dependent sumoylation is required to stabilize AGO1, which in turn acts to suppress the translation of the *IL6-HA* reporter in U2OS cells. This is in line with previous studies that have shown that the AGO1 protein strongly interacts with, and regulates the expression of *IL6* mRNA upon *let-7a* overexpression [28,29], and that RanBP2 is required for the *let-7*-mediated suppression of *luciferase* reporter mRNAs (Fig 5F and [41]).

### RanBP2 promotes the sumoylation, and inhibits the ubiquitination, of AGO1

When analyzing the overexpression of various versions of FH-AGO1, we noted that RanBP2-dE3 U2OS cells accumulated huge amounts of high molecular weight AGO1 that were often confined to the stacking gel (Fig 6G). This suggested that a significant proportion of FH-AGO1 accumulated post-translational modifications in RanBP2-dE3 cells, and raised the possibility that in the absence of RanBP2-dependent sumoylation, FH-AGO1 was poly-ubiquitinated and then targeted for decay. This high mobility AGO1 was not seen with the FH-AGO1^K564A/K568A^ mutant (Fig 6G), which does not bind to miRNAs, suggesting that RanBP2 may only affect the post-translational modification of active AGO1.

**Fig 7:**
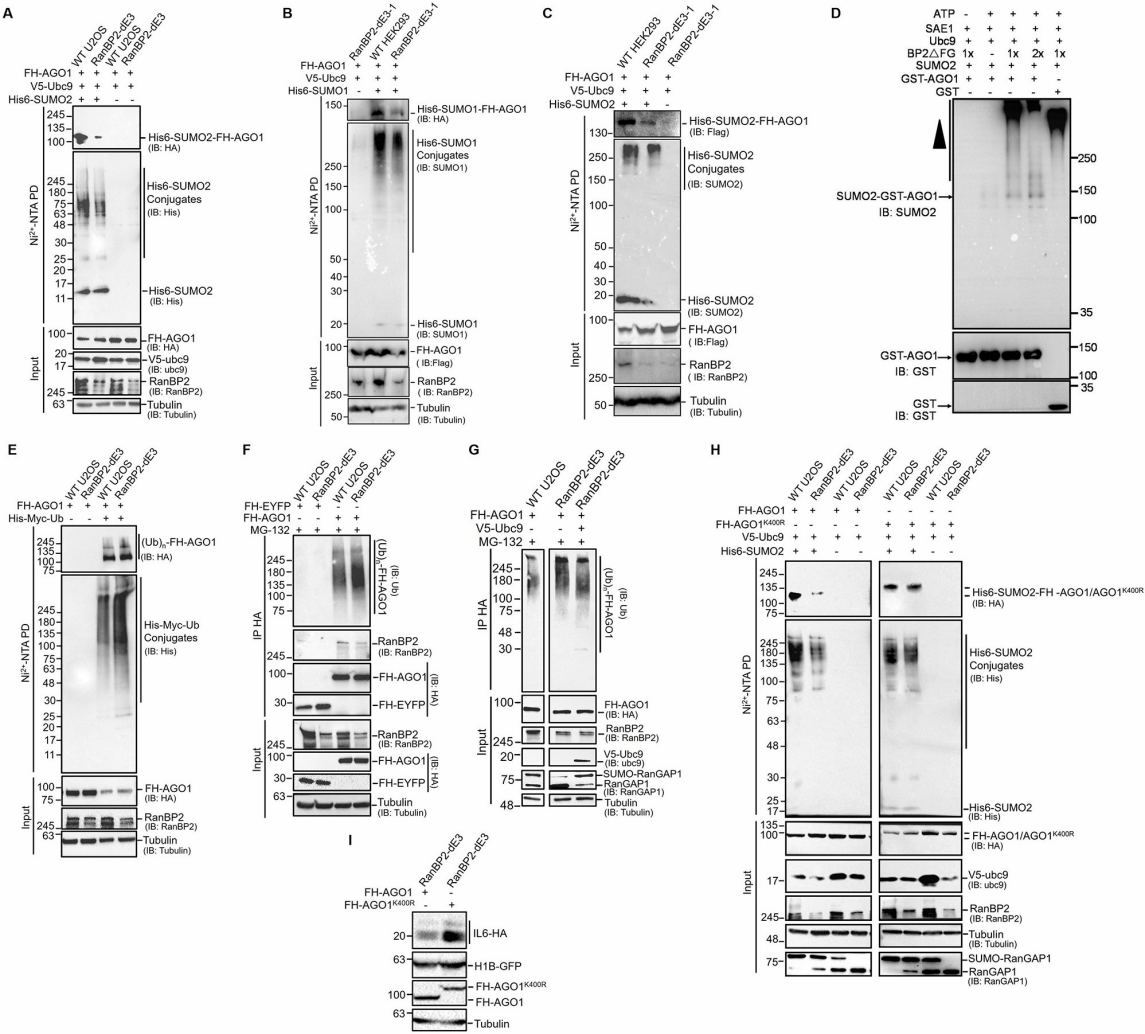
RanBP2 promotes the sumoylation and inhibits the ubiquitination of AGO1. (A) Unmodified and RanBP2-dE3 U2OS cells were co-transfected with plasmids expressing Flag-HA-tagged AGO1 (*FH-AGO1*), SV5-tagged Ubc9 (*V5-Ubc9*), and His-tagged SUMO2 (*His6-SUMO2* “+”) or control vector (*His6-SUMO2* “-”). 24 h post-transfection cells were lysed in 6 M Guanidinium-HCl, and the His6-SUMO2 conjugates were isolated on Nickel beads (“Ni^2+^ NTA PD”) or the lysates were directly analyzed (“input”) and separated by SDS-PAGE. Conjugates were analyzed for the presence of FH-AGO1 by immunoblotting for HA (IB: HA), and for total His6-SUMO2 conjugates by immunoblotting for His (IB: His). Input lysates were immunoblotted with antibodies against FH-AGO1, V5-Ubc9, RanBP2 and α-tubulin. See S10A Fig for the quantification of His6-SUMO2-FH-AGO1 levels. (B-C) As in (A), except that unmodified and RanBP2-dE3-1 HEK293 cells were transfected with *His6-SUMO1* (B) or *His6-SUMO2* (C). Antibodies used for immunoblotting were as indicated on the right. See S10B and S10C Fig for the quantification of His6-SUMO1-FH-AGO1 and His6-SUMO2-FH-AGO1 levels. (D) AGO1 was in vitro sumoylated with purified components, with SUMO2, active recombinant human RanBP2 protein (BP2ΔFG) as the SUMO E3-ligase, and recombinant GST-tagged human AGO1 protein (GST-AGO1) or GST as substrates. Equal amounts of SAE1, SUMO2, Ubc9, GST-AGO1 and GST (35 ng) were added to 10 μL reactions, where 1x is estimated to be 5 ng of BP2ΔFG. Negative controls lacking ATP or BP2ΔFG were also shown. After incubation at 30°C for 1 h, the reactions were analyzed by immunoblotting with antibodies indicated. The position of SUMO2-modified AGO1 is indicated and the SUMO2-conjugated BP2ΔFG is represented by the arrowhead. (E) Similar to (A), except that U2OS cells were co-transfected with plasmids for *FH-AGO1*, and His-Myc-tagged ubiquitin (*His-Myc-Ub* “+”) or control vector (*His-Myc-Ub* “-”). 18 h post-transfection, cells were treated with MG132 (10 μM) for an additional 7 h to preserve ubiquitinated conjugates. Cells were lysed in 6 M Guanidinium-HCl, and the His-Myc-Ub conjugates were isolated on Nickel beads (“Ni^2+^ NTA PD”) or the lysates were directly analyzed (“input”) and separated by SDS-PAGE. Conjugates were analyzed for the presence of FH-AGO1 by immunoblotting for HA (IB: HA), and for total His-Myc-Ub conjugates by immunoblotting for His (IB: His). See S10D Fig for the quantification of His-Myc-Ub-FH-AGO1 levels. (F) Unmodified and RanBP2-dE3 U2OS cells were transfected with plasmids for *FH-AGO1* or Flag-HA-tagged yellow fluorescent protein (*FH-EYFP*). 18 h post-transfection, cells were treated with MG132 (10 μM) for an additional 7 h to preserve ubiquitinated conjugates. Cells were lysed in RIPA buffer, and FH-AGO1/FH-EYFP and associated proteins were isolated by immunoprecipitation using anti-HA antibodies (“IP HA”) or the lysates were directly analyzed (“input”) and separated by SDS-PAGE. The immuoprecipitates were analyzed by immunoblotting for ubiquitinated proteins (IB: Ub), immunoprecipitated FH-AGO1/FH-EYFP by immunoblotting against HA, and for co-immunoprecipitated RanBP2. Input lysates were immunoblotted for RanBP2, FH-AGO1 and FH-EYFP (IB: HA) and α-tubulin. See S10E Fig for the quantification of (Ub)n-FH-AGO1 levels. (G) As in (F), except that unmodified and RanBP2-dE3 U2OS cells were co-transfected with FH-AGO1 and either V5-Ubc9 to enhance sumoylation or control plasmid. The immuoprecipitates were analyzed for ubiquitinated proteins by immunoblotting against ubiquitin (IB: Ub). Input lysates were immunoblotted for FH-AGO1 (IB: HA), RanBP2, V5-Ubc9, RanGAP1 and α-tubulin. Note that Ubc9 overexpression rescued RanGAP1-sumoylation in RanBP2-dE3 cells and decreased the amount of ubiquitinated FH-AGO1. See S10F Fig for the quantification of (Ub)n-FH-AGO1 levels. (H) As in (A), except that cells were transfected with either *FH-AGO1* or *FH-AGO1*^*K400R*^. Note that FH-AGO1^K400R^ was no longer sumoylated in a RanBP2-dependent manner. (I) RanBP2-dE3 U2OS cells were co-transfected with plasmids expressing an intron-containing IL6-HA construct (*IL6-1i-HA*), histone 1B-GFP (*H1B-GFP*) and either *FH-AGO1* or *FH-AGO1*^*K400R*^. Cell lysates were collected 24 h post-transfection, separated by SDS-PAGE and immunoblotted with antibodies against HA (IL6-HA, FH-AGO1 and FH-AGO1^K400R^), GFP and α-tubulin. See S11A Fig for the quantification of IL6-HA levels.

To identify whether RanBP2 promotes the sumoylation of AGO1, we co-expressed FH-AGO1 with His-tagged SUMO2 (His6-SUMO2). To enhance sumoylation, we co-transfected SV5-tagged Ubc9 (V5-Ubc9). Again, note that Ubc9 is the only known SUMO-conjugating E2 enzyme in humans. His6-SUMO2-conjugated FH-AGO1 was purified from denatured cell extracts (6 M Guanidinium-HCl) on a nickel column, which binds to the His tag on the exogenous SUMO2, and the eluate was analyzed using an anti-HA antibody. Note that since the lysate is denaturing, only proteins that were covalently bound to His6-SUMO2 were isolated. As we suspected, FH-AGO1 was strongly sumoylated in wildtype U2OS and relatively weakly in RanBP2-dE3 cells (Fig 7A, see quantification in S10A Fig). No FH-AGO1 was seen in the nickel-bound fraction from cells that did not express His6-SUMO2, indicating that the signal was specific to His6-SUMO2-FH-AGO1. When total His-tagged SUMO2 conjugated proteins were assessed, we saw a modest decrease in RanBP2-dE3 relative to wildtype U2OS cells (Fig 7A). Similarly, we observed that FH-AGO1 was strongly sumoylated (using either SUMO1 or SUMO2) in unmodified HEK293 cells and this decreased in RanBP2-dE3-1 HEK293 cells (Fig 7B and 7C, see quantification in S10B and S10C Fig).

To determine whether RanBP2 could directly sumoylate AGO1, we set up an in vitro sumoylation assay with purified components, including GST-AGO1, SUMO2, E1 (SAE1), E2 (Ubc9) and the RanBP2 E3 domain (BP2ΔFG). We observed a sumoylated band at ~140 kDa that corresponded to SUMO2-GST-AGO1 (Fig 7D, lanes 3 and 4). The presence of this SUMO2-positive band was dependent on ATP (lane 1) and BP2ΔFG (lane 2). GST was not detectably sumoylated in this reaction (lane 5). High molecular weight SUMO2-positive bands likely represent the auto-sumoylation of BP2ΔFG, as reported by other groups [2].

Next, we wanted to assess the effect of the RanBP2 mutation on AGO1 ubiquitination. We co-expressed FH-AGO1 with His-Myc tagged ubiquitin (His-Myc-Ub) in unmodified and RanBP2-dE3 U2OS cells. To ensure that we would capture ubiquitinated intermediates, we inhibited protein degradation with MG132. We then purified ubiquitinated substrates from the cell lysates on a nickel column, which binds to the His-tag on the exogenously expressed ubiquitin. In contrast to what we had seen with sumoylation, the level of ubiquitinated FH-AGO1 was 30% higher in RanBP2-dE3 cells compared to wildtype U2OS (Figs 7E and S10D). Again, no FH-AGO1 was seen in the nickel-bound fraction from cells that did not express His-Myc-Ub, indicating that the signal was specific. Furthermore, when total ubiquitinated products were blotted for, using antibodies against the His tag, we saw a general increase in ubiquitinated substrates, indicating that the RanBP2-dE3 cells had higher levels of ubiquitinated proteins.

To confirm this last result, we immunoprecipitated exogenously expressed FH-AGO1 from MG132-treated cell lines and blotted for endogenous ubiquitin to visualize ubiquitinated conjugates. Again, the level of ubiquitinated AGO1 increased in RanBP2-dE3 cells by about 60% compared to wildtype U2OS (Figs 7F and S10E), even though equal amounts of FH-AGO1 were assessed (Fig 7F). Immunoprecipitated FH-EYFP did not display any ubiquitinated conjugates (Fig 7F), showing that ubiquitination did not occur on any overexpressed protein.

We next want to ensure that the reason that RanBP2-dE3 cells promoted an increase in ubiquitination was due to the loss of its ability to sumoylate downstream targets, and not some other activity that happened to be disabled by the RanBP2-dE3 mutations, such as binding to RanGAP1. To increase the overall levels of sumoylation in these cells, we overexpressed the V5- Ubc9. Indeed, V5-Ubc9 overexpression resulted in an increase in sumoylated RanGAP1 (Fig 7G), supporting the notion that this protocol did indeed result in an increase in overall sumoylation activity in RanBP2-dE3 cells. When FH-AGO1 was immunoprecipitated and the level of its ubiquitin-conjugates was assessed by immunostaining, we observed that V5-Ubc9 overexpression reduced overall ubiquitination in RanBP2-dE3 cells (Figs 7G and S10F). Thus, the overexpression of Ubc9, and the accompanying general increase in sumoylation activity, reduced FH-AGO1 ubiquitination even in RanBP2-dE3 cells.

From these experiments we conclude that RanBP2 promotes the sumoylation of AGO1 and inhibits its ubiquitination. In addition, our experiments also suggest that sumoylation inhibits ubiquitination of AGO1 in U2OS cells.

### RanBP2 associates with AGO1

Previously it had been reported that RanBP2 directly interacts with AGO2 [41]. Indeed, we found both RanBP2 and the RanBP2-dE3 mutant in immunoprecipitates of FH-AGO1 (Fig 7F), but not in control immunoprecipitates (FH-EYFP). This suggests that RanBP2 interacts with AGO1 in a manner analogous to AGO2. The finding that RanBP2 directly binds to AGO1 is consistent with the idea that RanBP2 directly sumoylates Argonaute proteins.

Interestingly, it was previously reported that this interaction required one of two putative SUMO-interacting motifs (SIMs) that are present in the E3 domain of RanBP2 [41]. Although these mutant cells have versions of RanBP2 that have mutations that disrupt the first SIM, the second putative SIM is still intact (S3 Fig). While this interaction would suggest that RanBP2 should bind to sumoylated Argonautes, Sahoo and colleagues clearly detected interactions between RanBP2 and unmodified AGO2. This is also consistent with our findings that despite the fact that AGO1 in RanBP2-dE3 U2OS cells is poorly sumoylated (see Fig 7A), it still associates with RanBP2 at levels close to what is seen in unmodified cells (Fig 7F).

### Mutations of lysine 400 in AGO1 suppress its sumoylation, its turnover and its ability to downregulate IL6

Several studies have identified lysine 402 of AGO2 as a RanBP2-dependent sumoylation site [35,37]. To determine whether AGO1 was sumoylated at the cognate site, we tested a lysine-to-arginine mutant (FH-AGO1^K400R^) in the in vivo sumoylation assay in U2OS cells. Indeed, this mutant was no longer sumoylated in a RanBP2-dependent manner, although sumoylated forms could still be detected (Fig 7H). The mutant also did not suppress the production of IL6-HA protein as well as wildtype FH-AGO1 (Figs 7I and S11A). Intriguingly, the mutant protein consistently migrated at a higher molecular weight when compared to the non-mutant form, suggesting that it is constitutively modified, although the nature of this modification remains unclear (Fig 7I). Consistent with the idea that the K400R mutation disrupts the regulation of AGO1 decay, we observed that this mutant is stable in both unmodified and RanBP2-dE3 U2OS cells (S11B and S11C Fig).

These results are consistent with the idea that lysine 400 is a key regulatory motif for AGO1. Our results suggest that this residue is sumoylated in a RanBP2-dependent manner and that this is required for its ability to silence the *IL6* mRNA. Furthermore, the lack of decay of this mutant in RanBP2-dE3 cells may indicate that this residue is not only the site of sumoylation, but also ubiquitination. Despite this, we cannot rule out the possibility that the K400R mutation either directly, or indirectly (by triggering a modification), inhibits AGO1 activity.

### In HAP1 and HEK293 cells, RanBP2 regulation of IL6 translation is not accompanied by AGO1 destabilization

**Fig 8:**
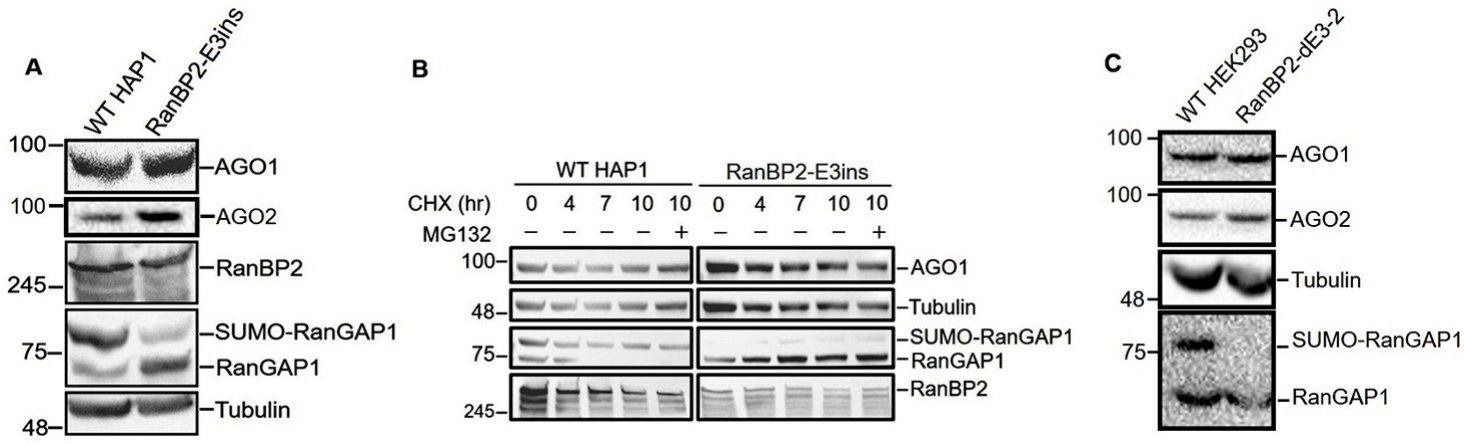
RanBP2 SUMO E3-ligase activity is not required for the stability of AGO1 in HAP1 and HEK293 cell lines. (A) Unmodified and RanBP2-E3ins HAP1 cells were lysed, separated by SDS-PAGE, and immunoblotted with antibodies against AGO1, AGO2, RanBP2, RanGAP1, and α-tubulin. (B) Unmodified HAP1 and RanBP2-E3ins cells were treated with cycloheximide (CHX, 100 μM) for various amounts of time to block further translation and with or without proteasome inhibitor MG132 (50 μM) (MG132 “+” or “-”). Cell lysates were collected, separated by SDS-PAGE, and immunoblotted with antibodies against AGO1, α-tubulin, RanGAP1 and RanBP2. (C) As in (A), except for unmodified and RanBP2-dE3-2 HEK293 cells.

To validate our results, we next tested whether AGO1 stability was compromised in HAP1 cells. To our surprise, HAP1-RanBP2-E3ins cells did not show lower levels of AGO1 compared to unmodified HAP1 cells (Fig 8A). We next validated our results by measuring AGO1 turnover in cycloheximide-treated cells. Indeed, AGO1 levels did not significantly drop in either unmodified or RanBP2-E3ins HAP1 cells (Fig 8B). Furthermore, MG132-treatment did not lead to an increase in AGO1 in these cells over the CHX-time course (Fig 8B), suggesting that this protein is very stable in this mutant cell line. Similarly, HEK293-RanBP2-dE3-2 cells also did not show a reduction in either AGO1 or AGO2 compared to unmodified cells (Fig 8C).

From these results, we concluded that despite the fact that IL6-silencing is impaired in HAP1 and HEK293 cells when RanBP2-dependent sumoylation is inhibited, this was not due to a decrease in AGO1 stability. Rather, it would seem, that RanBP2-dependent sumoylation is affecting some other aspects of AGO1-mediated silencing. In some cases (U2OS cells), this is accompanied by AGO1 degradation, while in other cases (HAP1 and HEK293 cells), it is not. This also raised the possibility that AGO2 may also be involved in silencing of the *IL6* mRNA. Although it does not require RanBP2 for its stability in any of the tested cell lines, its activity may require sumoylation.

### Argonautes associate with *IL6* mRNA in the nucleus

**Fig 9:**
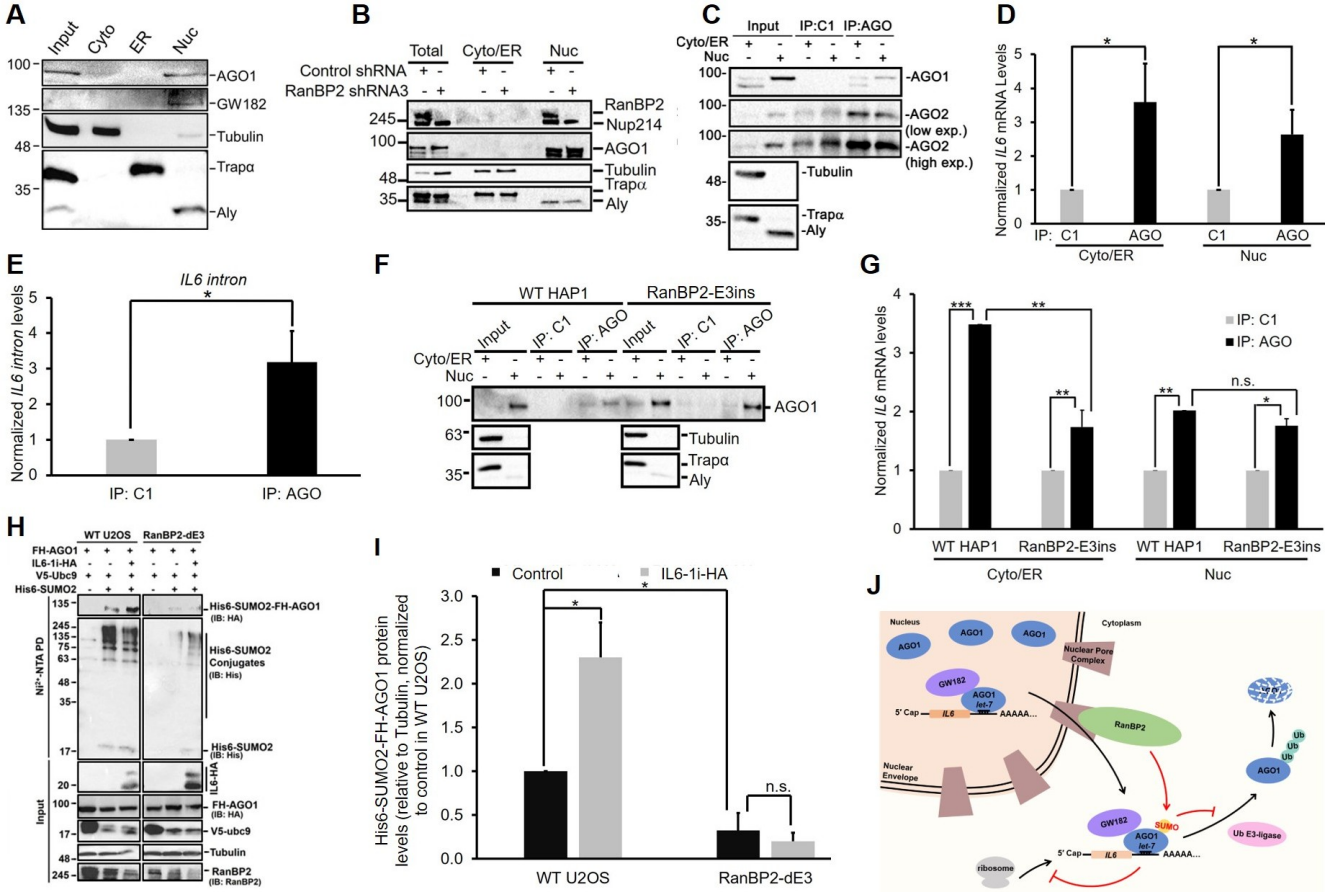
Nuclear Argonautes interact with *IL6* mRNA, and require RanBP2-dependent sumoylation to maintain their association with mRNA in the cytosol. (A) U2OS cells were either directly lysed in RIPA buffer (“Input”) or fractionated into cytoplasmic (“Cyto”), ER and nuclear (“Nuc”) fractions. Samples were separated by SDS-PAGE and immunoblotted for AGO1, GW182, α-tubulin (cytosolic marker), Trapα (ER marker) and Aly (nuclear marker). (B) Similar to (A), except that U2OS cells were infected with lentivirus that delivered shRNA3 against RanBP2 for four days, and were then fractionated into cytoplasmic/ER and nuclear fractions. Samples were analyzed by immunoblotting with antibodies against mAb414 (RanBP2 and Nup214), AGO1, α-tubulin (cytosolic marker), Trapα (ER marker) and Aly (nuclear marker). (C-D) U2OS cells were transfected with intronless IL6-HA construct (*IL6-Δi-HA*). 24 h post-transfection, cells were fractionated into cytoplasmic/ER and nuclear fractions. Fractions were either collected (“Input”) or immunoprecipitated with control (C1) or anti-AGO synthetic antibody fragment. Samples were separated by SDS-PAGE and immunoblotted for AGO1, AGO2 (shown in both low and high exposure), α-tubulin, Trapα and Aly (C). The amount of *IL6* mRNA in the immunoprecipitates was quantified by RT-qPCR, each bar representing the average of four independent experiments ± SEM (D). (E) U2OS cells were transfected with an intron-containing IL6-HA construct (*IL6-1i-HA*). 24 h after transfection, cells were lysed and immunoprecipitated with control (C1) or anti-AGO synthetic antibody fragment. The amount of *IL6 intron* in the immunoprecipitates was quantified by RT-qPCR, each bar being the average of five independent experiments ± SEM. (F-G) Similar to (C-D), except for unmodified and RanBP2-E3ins HAP1 cells. Each bar is the average of three independent experiments ± SEM. (H-I) Unmodified and RanBP2-dE3 U2OS cells were co-transfected with plasmids for Flag-HA-tagged AGO1 (*FH-AGO1*), SV5-tagged Ubc9 (*V5-Ubc9*), with or without His-tagged SUMO2 (*His6-SUMO2* “+/-”) and an intron-containing IL6-HA construct (*IL6-1i-HA* “+/-”). 24 h post-transfection, cells were lysed in 6 M Guanidinium-HCl, and the His6-SUMO2 conjugates were isolated on Nickel beads (“Ni^2+^ NTA PD”) or the lysates were directly analyzed (“input”) and separated by SDS-PAGE. Conjugates were analyzed for the presence of FH-AGO1 by immunoblotting for HA (IB: HA), and for total His6-SUMO2 conjugates by immunoblotting for His (IB: His). Input lysates were immunoblotted with antibodies as indicated (H). Isolated His6-SUMO2-FH-AGO1 and α-tubulin levels were quantified using densitometry analysis and the ratio of His6-SUMO2-FH-AGO1/α-tubulin was normalized to unmodified U2OS cells without IL6-1i-HA. Each bar is the average of two independent experiments ± SEM. (J) General model for how RanBP2 regulates the silencing of the *IL6* mRNA through the sumoylation of AGO1. *P = 0.01–0.05, **P = 0.001–0.01, ***P < 0.001, n.s. indicates no significant difference (Student’s *t*-test).

Our results in HAP1 and HEK293 cells raised the possibility that RanBP2-dependent sumoylation had some other effects on AGO1 activity and that in addition AGO1 became unstable in U2OS cells. Interestingly, we observed that AGO1 and GW182 were both primarily localized to the nucleoplasm of U2OS cells (Fig 9A). This was also true for AGO2 (Fig 9C, see the input lanes in the AGO2 immunoblot, high exposure). We found similar results when we assessed AGO1 and AGO2 localization in unmodified and RanBP2-E3ins HAP1 (S12A Fig) and in HEK293 cells (S12B Fig). This is in contrast to overexpressed FH-AGO1, where only a fraction of the protein is found in the nucleus as assessed by immunofluorescence and fractionation (S13 Fig). The association of AGO1 with the nuclear fraction was unlikely due to its tethering to the nuclear pore by RanBP2, as depletion of the later by lentiviral delivered shRNA did not affect the nuclear localization of the former (Fig 9B–note that cells were treated with MG132 to maintain AGO1 levels). Our observations that AGO1 and AGO2 are primarily nuclear are consistent with other reports that have indicated that endogenous RISC complexes are found in the nuclei of several tissue culture cell lines and in cells derived from various animal tissues [53–58]. The fact that at steady state most of the *IL6* mRNA is in the cytosol (Fig 1G), yet not being translated, must mean that endogenous AGO1 silences the cytoplasmic pool of mRNA. These observations raised the possibility that endogenous Argonautes are recruited to the *IL6* mRNA in the nucleus and that upon the completion of nuclear export, AGO1 (and perhaps AGO2) becomes sumoylated by RanBP2 as part of an mRNP maturation process. The sumoylation of Argonautes would then be required for their stable association to the *IL6* mRNA and promote silencing. If AGO1 is not sumoylated, this would lead to its dissociation from *IL6* mRNA and result in AGO1 decay in U2OS cells, or cytoplasmic accumulation of free AGO1 in HAP1 and HEK293 cells (see model in Fig 9J).

To test this model, we wanted to determine whether endogenous nuclear Argonautes could associate with *IL6* mRNA. We thus used a synthetic antibody [59] to immunoprecipitate endogenous Argonautes (both AGO1 and AGO2) from cytosolic and nuclear fractions of U2OS cells expressing *IL6-Δi-HA* (Fig 9C) and tested for the co-precipitation of *IL6* mRNA by RT-qPCR. As predicted by our model, *IL6* mRNA was enriched in the immunoprecipitated nuclear Argonaute fraction, as well as the cytoplasmic fraction (Fig 9D). Moreover, when we repeated the experiment with cells expressing intron-containing *IL6-HA* reporter, we observed that endogenous AGO1/AGO2 immunoprecipitates were enriched for *IL6* intron (Fig 9E), strongly indicating that Argonautes bind to pre-mRNAs, which are expected to be restricted to the nucleus. We thus conclude that Argonautes (AGO1 and/or AGO2) can associate with mRNAs in the nucleus.

### RanBP2-dependent sumoylation selectively disrupts the association of Argonautes with *IL6* in the cytoplasm and not in the nucleus

According to our model, Argonautes should associate with newly synthesized *IL6* mRNAs in the nucleus, and upon completion of nuclear export, the further maintenance of the Argonaute-*IL6* complex should require RanBP2-dependent sumoylation. Since the levels of endogenous AGO1 fluctuate in a RanBP2-dependent manner in U2OS cells, we decided to test this idea in HAP1 cells, where AGO1 levels do not drastically change between unmodified and RanBP2-mutant cell lines (Fig 8A). We thus immunoprecipitated Argonautes from the cytoplasmic and nuclear fractions of unmodified and RanBP2-E3ins HAP1 cells expressing *IL6-Δi-HA* (Fig 9F) and tested for the co-precipitation of *IL6* mRNA by RT-qPCR. As predicted by our model, nuclear Argonaute immunoprecipitates from both cell lines had similar levels of *IL6* mRNA (Fig 9G). In contrast, cytoplasmic Argonaute immunoprecipitates from RanBP2-E3ins HAP1 cells had significantly lower levels of *IL6* compared to immunoprecipitates from unmodified cells (Fig 9G).

### Expression of *IL6* mRNA promotes the sumoylation of AGO1

Our model predicts that the overexpression of *IL6* mRNA should drive more RISC through the nuclear pore and hence increase the sumoylation of AGO1. In agreement with this, we observed that U2OS cells expressing IL6 had higher levels of sumoylated FH-AGO1 (Fig 9H and 9I). This was dependent on RanBP2, as the increase in sumoylation was not seen in RanBP2-dE3 U2OS cells (Fig 9H and 9I).

In summary, our data supports our model that Argonautes are loaded onto the *IL6* mRNA in the nucleus, and that upon the completion of nuclear export, AGO1 (and likely AGO2) is sumoylated by RanBP2. This post-translational modification likely stabilizes AGO1 onto the mRNA thus enforcing silencing (Fig 9J). In the absence of RanBP2-dependent sumoylation, Argonautes no longer remain associated with *IL6* mRNA, and in U2OS cells this triggers their ubiquitination and degradation.

## Discussion

In this study, we demonstrate that the nuclear pore filament protein RanBP2, sumoylates the *IL6* mRNP component AGO1, and likely AGO2, which ultimately promotes the silencing of the *IL6* mRNA. Our data is consistent with the idea that Argonautes are recruited to the mRNP in the nucleus and are sumoylated just after the *IL6* mRNP complex completes export as part of an mRNP maturation event. This sumoylation is required for Argonaute to be retained in the mRNP and for its silencing activity. This model is supported by our data and observations made by other groups.

First, we show that in the three cell lines that we have investigated, endogenous Argonautes are primarily found in the nucleus (Figs 9A and S12). This localization is not dependent on RanBP2 (Fig 9B). Second, we demonstrate that nuclear Argonautes associate with *IL6* mRNA (Fig 9D and 9G), and similar results have been documented by other groups [53–58]. Thus, it is likely that in these cell lines most mRNA-Argonaute complexes are initially formed in the nucleus rather than in the cytoplasm where Argonaute levels appear to be very low at steady state. Third, this association in the nucleus does not require RanBP2-dependent sumoylation (Fig 9G), indicating that RanBP2 likely acts downstream from the initial mRNA-AGO/miRNA binding event. Fourth, several groups have noted that Argonaute-mRNA complexes are very stable and have long half-lives [60,61], suggesting that once formed in the nucleus, these mRNA-Argonaute complexes should persist as the mRNP is exported to the cytoplasm, unless they are actively disrupted. Fifth, we show that the overexpression of *IL6* mRNA drives the sumoylation of AGO1 (Fig 9H and 9I), suggesting that it is the mRNP-associated AGO1 that is sumoylated by RanBP2. In the absence of RanBP2-dependent sumoylation, Argonautes are likely evicted from the mRNP (Fig 9G), and at least in U2OS cells, are ubiquitinated and degraded by the proteosome. In HEK293 cells, AGO1 is also sumoylated (Fig 7B and 7C) and this modification is likely required to maintain the Argonaute-mRNA association in the cytoplasm, as we documented in HAP1 cells (Fig 9G). The evicted AGO1 is then likely recycled back into the nucleus in these cell lines. This is also likely the case for AGO2 in U2OS, HEK293 and HAP1 cells. The idea that only mRNA-bound Argonautes are subjected to post-translational modifications is supported by the observation that a mutant form of AGO1 that lacks miRNA-binding capability (FH-AGO1^K564A/K568A^) is not modified (likely by poly-ubiquitination) in cells that lack RanBP2-dependent SUMO-ligase activity (Fig 6G). Although it is possible that these two lysines may be modified (by either SUMO or ubiquitin), they lie within the interior of the protein and are likely not readily accessible to modification enzymes. Finally, it is clear from our data that *IL6* mRNA is translationally repressed in the cytoplasm in a manner that requires RanBP2-dependent sumoylation (Fig 3B) and this is in line with previous reports that have demonstrated that RanBP2 is required for the stable association of AGO2 with target mRNAs [41].

We cannot entirely rule out the possibility that Argonautes are able to initiate interactions with *IL6* mRNA in the cytoplasm, and that these mRNPs then travel to the nuclear pore to become sumoylated by RanBP2, although this type of mRNP trafficking has not been reported in the literature. Alternatively, mRNPs containing AGO1 may visit annulate lamelli, which consists of stacks of ER that contain nuclear pores, as previously suggested [41]. Although it is possible that some mRNPs encounter these structures, our data suggests a much more plausible scenario as AGO1 levels are highest in the nucleus, thus favoring interactions with target mRNAs soon after their synthesis. Since these mRNPs must cross the nuclear pore prior to entering the cytoplasm, this would guarantee that all of the mRNA-associated Argonautes would be in the vicinity of RanBP2 at some point and thus ensure that silencing is enforced in the cytoplasm. Indeed, in single molecule live cell imaging experiments, it has been noted that upon the completion of export, most mRNPs have extended dwell times on the cytoplasmic face of the nuclear pore complex, right where RanBP2 is located [4].

We have documented that when tagged Argonautes are overexpressed, they build up in the cytoplasm (S13 Fig) and silence mRNAs in cells that lack RanBP2-dependent sumoylation (Fig 6E). In light of these observations, other reports that have examined how overexpressed tagged-AGO1 or AGO2 associate with mRNAs may have overlooked the importance of nuclear-cytoplasmic trafficking of Argonautes. Indeed, it has been shown that miRNA-bound Argonautes traffic to the nucleus and require importins to effectively silence their target mRNAs [62].

Our study is one of the first investigating how mutations in RanBP2 may contribute to the development of ANE1. Our results indicate that RanBP2 regulates the expression of two ANE1-associated cytokines, IL6 and TNF-α, and suggest that these may be altered in ANE1 patients. In agreement with our findings, it is well known that *let-7* is a major regulator of the *IL6* mRNA [28,29] and that this miRNA regulates inflammatory signalling [33,34]. Moreover, there is evidence that *let-7* levels change in response to infection [29–32]. From all these data, an overall model emerges where upon infection, *let-7* and RanBP2 modulate the inflammatory response by downregulating IL6, and potentially other cytokines. A defect in this regulation may cause a hyperinflammatory response that leads to pathology. Importantly, the overproduction of only one or two of these cytokines may ultimately result in the activation of autocrine and paracrine loops that lead to the overproduction of many other cytokines. Significantly, we found that a version of RanBP2 that contains 3 of the ANE1-point mutations is still able to silence *IL6* mRNA. In many ways this is not surprising. First, these mutations are only 40% penetrant, indicating that there may be other confounding variables at play in contributing to the misregulation of cytokines. Second, the miRNA pathway is essential for proper development in mammalians [63], and we would expect that the miRNA pathway would be operational in most ANE1 patient as these individuals lead essentially normal lives until they experience a viral infection. It is possible that these mutations only matter in virally infected cells, perhaps by modifying how RanBP2 interacts with either anti-viral host proteins or viral proteins. Indeed, many viruses produce proteins that interact with nuclear proteins [64]. Interestingly, COVID-19 also causes a cytokine storm, except that unlike in ANE1, this is typically confined to the lungs [65]. Despite this, several COVID-19 patients have displayed ANE-like symptoms [65–67]. Thus, it is likely that a similar dysregulation of cytokine expression may explain why COVID-19 causes death in a subset of patients. Indeed, several SARS-Cov2 proteins interact with components of the nuclear pore [68] and our results suggest that these interactions may contribute to the misregulation of cytokine mRNAs.

Interestingly, many small RNA pathways (i.e. germ granule small RNAs in *Caenorhabditis elegans* and piRNAs in *Drosophila*, zebrafish and mouse) involve the processing and/or loading of small RNAs onto their target complexes in large phase-separated structures, called nuages or germ granules, that are physically associated with nuclear pores [69]. Indeed, a recent report demonstrated that RanBP2 was required for piRNA silencing of transposable elements in *Drosophila* [70]. These studies lend support to our model that RanBP2 may help to assemble or simply stabilize Argonautes onto target mRNAs, after they emerge from the nuclear pore.

Argonautes are known to be extensively post-translationally modified, especially by ubiquitination [35–40,71,72]. Our data indicates that RanBP2 directly sumoylates AGO1, as it has been previously reported for AGO2 [35,37], and that this sumoylation antagonizes AGO1 ubiquitination in U2OS cells. How this would work is unclear at the moment. Sumoylation of a particular lysine residue may prevent the same residue from being ubiquitinated and thus stabilize the protein, as reported in other cases [73]. Alternatively, sumoylation of AGO1 may mask the binding site of a ubiquitin E3-ligase or help recruit a ubiquitin protease. Interestingly, the ubiquitination site of *Drosophila* AGO1 (the homolog of human AGO2), K514, which is recognized by the RING-type ubiquitin E3-ligase Iruka [40], is not conserved within human AGO1, suggesting that AGO1 and AGO2 may have different modes of regulation. These possibilities can be sorted out by mapping the relevant ubiquitinated residues on AGO1 and determining whether mutating these residues disrupt RanBP2-dependent regulation. Uncovering the ubiquitin E3-ligase would help clarify these issues.

## Materials and methods

### Plasmid constructs

For all expression experiments, pCDNA3.1 plasmid containing the human *insulin* cDNA [12] and human *β-globin* [74]; pEGFP plasmid containing the *H1B-GFP* fusion gene [75] were described previously. The *interleukin 6* (*IL6*) gene cloned inside pSPORT6 plasmid was purchased from OpenBiosystems, and various versions of the *IL6* gene including *MHC-IL6-Δi*, *IL6-1f*, *IL6-1i*, *5F-IL6-1i*, *5βG-IL6-1i*, *IL6-1i-3F*, *IL6-1i-3del1*, *IL6-1i-3del2*, and *IL6-1i-Let7m*, were cloned inside the pcDNA3.1 plasmid and associated mutations were made by restriction-enzyme cloning or site-directed mutagenesis (according to manufacturer’s protocol). pIRESneo-*FLAG/HA-AGO1* (Addgene plasmid # 10820, FH-AGO1) and pIRESneo-*FLAG/HA-EYFP* (Addgene plasmid # 10825, FH-EYFP) were gifts from T.Tuschl [51]. The AGO1 site mutant construct FH-AGO1^K564A/568A^ was constructed from FH-AGO1 by restriction-enzyme-free cloning [76] using the following primer sequences, K564A/568A-forward primer: 5′-ATGTCGCACTTGGTGGCATTAACAACATCCTAG-3′ and K564A/568A-reverse primer: 5′-TGATCGCGAGGCAGAGGTTGGACAGAGTCTG-3′. The human *His6-SUMO1*, *His6-SUMO2* (in pcDNA3), *V5-Ubc9* and *His-Myc-ubiquitin* plasmid were gifts from L. Frappier [77–79]. shRNA plasmids (details below) were purchased from Sigma; the *CRISPR/CAS9* plasmid, pSpCas9(BB)-2A-Puro (PX459) V2.0 was a gift from F. Zhang (Addgene plasmid # 62988) [80], and plasmids of Dual Luciferase Reporter Assay (psiCHECK2-Luc-IL6 and psiCHECK2-Luc-IL6-(Let7m), details below) were gifts from J. Vogel [29]. TNF-α (ORF and UTR) was first amplified from U2OS genomic cDNA and then cloned into pcDNA3. The HA tag was then inserted at the 3′ end of the ORF by restriction-enzyme-free cloning.

### Cell culture, cell transfection and lentiviral mediated shRNA knockdown

Cell culture and transfection were carried out as described previously [5,81]. Briefly, both human osteosarcoma (U2OS) and embryonic kidney 293 (HEK293) were maintained in Dulbeco’s Modified Eagle Medium (DMEM) supplemented with 10% fetal bovine serum, and 1% penicillin-streptomycin (WISENT). HAP1 cells (a gift from Alexio Muise) can be obtained from Horizon Genomics (Vienna, Austria). HAP1 cells were grown in Iscove’s modified Dulbecco’s medium (IMDM) supplemented with 10% fetal bovine serum and 1% penicillin-streptomycin (WISENT). All cells were cultured at 37°C in a 5% CO_2_-humidified incubator. For chemical treatments, MG132 (Sigma) and cycloheximide (CHX) (Sigma) were dissolved in DMSO or in ethanol and used at a final concentration of 10 μM and 100 μM, respectively.

All cells were plated 24 h before transfection and transfected at a confluency of 70–80% using GenJet-OS in vitro transfection reagent for U2OS cells (SignaGen Laboratories, Gaithersburg, MD, USA) or JetPRIME for HEK293 cells (PolyPlus) or Turbofectin 8.0 for HAP1 cells (OriGene), following the manufacturer’s protocol.

Lentiviral-mediated shRNA knockdown was carried out as described previously [82] with plasmids encoding shRNA against RanBP2 (shRNA1: TRCN0000003452, shRNA3: TRCN0000003454, Sigma), AGO1 (shRNA: TRCN0000007859, Sigma), AGO2 (shRNA: TRCN0000011203, Sigma), Dicer (shRNA: TRCN0000051258, Sigma), GW182 (shRNA: TRCN0000376423, Sigma), Ubc9 (shRNAs: TRCN0000320448, TRCN0000368347), or control vector (pLK0.1). Briefly, plasmids encoding shRNA were transfected into the HEK293 cells together with the accessory plasmids, VSVG and △8.9, to generate lentivirus carrying specific shRNA plasmids. Lentivirus was harvested from the medium 48 h post-transfection by filtering through a 0.44 μm filter. For infection, lentivirus was applied to U2OS cells with 8 μg/ml hexadimethrine bromide. Puromycin was applied to the cell 24 h post-infection at 2 μg/ml to select for infected cells, and puromycin containing medium was changed every other day. Cell lysates were collected 5 days post-infection to assess the level of knockdown, and the cells were used for various experiments as described.

### Immunoblotting and immunoprecipitation

For immunoblotting, various culture cell lines were lysed with lysis buffer containing 50 mM Tris-HCl, 150 mM NaCl, 1% Triton X-100, 1 mM EDTA, and complete protease inhibitor cocktail (Roche), pH 7.4, on ice for 30 min. For immunoprecipitation, whole-cell extracts were collected 24–48 h after transfection and lysed in lysis buffer on ice for 30 min. After centrifugation for 30 min at 13,000 *g*, 4°C, supernatants were collected and incubated with Protein-G Sepharose beads coupled to specific antibodies (2 μg per pulldown) for 2–3 h with rotation at 4°C. The beads were washed 3 times with lysis buffer and bound proteins were eluted by boiling for 10 min in sample buffer containing 50 mM Tris-HCl (pH 6.8), 2% SDS, 10% glycerol, 0.1% bromophenol blue and 1% β-mercaptoethanol. For immunoblot analysis, immunoprecipitates or whole-cell lysates were separated by SDS-PAGE, transferred to nitrocellulose membrane and probed with primary antibodies against HA (HA-7 mouse monoclonal, 1:2000 dilution, Sigma; or rabbit polyclonal, 1:1000 dilution, Sigma), α-tubulin (mouse monoclonal DM1A, 1:1000 dilution, Sigma), GFP (rabbit polyclonal, 1:1000 dilution, Invitrogen), RanGAP1 (mouse monoclonal, 1:1000 dilution, Santa Cruz), RanBP2 (mouse monoclonal mAb414 1:5000 dilution, Cederlane, rabbit polyclonal, 1:1000 dilution, Abcam, or mouse monoclonal, 1:200 dilution, Santa Cruz), Ubc9 (rabbit polyclonal, 1:1000, Cell Signaling), IL6 (mouse monoclonal, 1:2000 dilution, Abcam), AGO2 (11A9 rat monoclonal, 1:1000 dilution, Millipore), His (mouse monoclonal, 1:1000 dilution, Abcam), AGO1 (rabbit monoclonal, 1:1000 dilution, Cell Signalling), ubiquitin (rabbit polyclonal, 1:500 dilution, Dako), GAPDH (rabbit polyclonal, 1:1000 dilution, ABGENT), GW182 (rabbit polyclonal, 1:1000 dilution, Abcam), Dicer (rabbit polyclonal, 1:1000 dilution, Cell Signaling), Trapα (rabbit polyclonal, 1:1000 dilution, [83]), or Aly (rabbit polyclonal, 1:1000 dilution, [84]). Subsequently, the relevant horse radish peroxidase (HRP) conjugated anti-rabbit (1:2000, Cell Signaling), anti-mouse (1:4000, Cell Signaling), anti-rat (1:3000, Cell Signaling) secondary antibody was used. Chemiluminescence luminol reagent (Pierce) and the Versadoc system (Bio-Rad) were used to visualize the blots. ImageJ (NIH) was used for densitometry analysis.

### Fluorescent in situ hybridization (FISH) and immunofluorescence microscopy

Fluorescence in situ hybridization (FISH) staining was done using DNA specific probes against *IL6* (GTAACATGTGTGAAAGCAGCAAAGAGGCACTGGCAGAAAACAACCTGAAC, 5′ labelled with Alexa546, IDT) at a dilution of 1:500 in 60% formamide hybridization buffer as previously described [81]. Samples were mounted on Fluoromount with 4’,6-diamidino-2-phenylindole (DAPI) (Southern Biotechnologies, Birmingham, AL, USA). Immunofluorescence staining was performed as previously described [81,82] using antibody against RanGAP1 (mouse monoclonal, 1:250 dilution, Santa Cruz), RanBP2 (rabbit polyclonal, 1:1000 dilution, Abcam), FLAG (M2 mouse monoclonal, 1:1000 dilution, Sigma) and a secondary antibodies (Alexa647/Alexa568/Alexa488-conjugated donkey anti-mouse/rabbit polyclonal; 1:1000; Life Technologies, Carlsbad, CA, USA). Microscopy, imaging, nuclear mRNA export quantifications and protein nuclear/cytoplasmic distribution quantifications were performed as previously described [5,12,81]. An epifluorescence microscope on a TI-E inverted Nikon microscope using a 60X phase 2, oil objective and a Coolsnap HQ2 14 bit CCD camera (photometric, Tucson, AZ, USA) controlled using NIS elements Basic Research Microscope Imaging Software (2009) was used to capture all the images. Image exposures varied from 30 ms to 2 s. Data pertaining to total integrated intensity, cytoplasmic/total, and nuclear/total fluorescence intensity was calculated as previously described [81] from raw, unprocessed images. Images shown in figures were adjusted for brightness and contrast using the Photoshop (Adobe).

### RNA isolation and northern blotting

After 18–24 h of transfection, total RNA from cultured cells was extracted with Trizol Reagent (Invitrogen) according to the manufacturer’s protocol. RNA was separated on a denaturing agarose gel, transferred, and probed for *IL6* and *Tubulin* as previously described [5].

### Generation of the SUMO E3 domain mutants of RanBP2 by CRISPR/Cas9

Genome editing of RanBP2 in U2OS, HEK293 or HAP1 cells was performed as previously described [85]. In brief, gRNA-dE3-1# (5′-GGGCTTTCTGCTCAGCGGT-3′), or gRNA- dE3-3# (5′-TGTAGCAGAAGAAAGTTGG-3′) that targets exon 21 of RanBP2 (Figs 2A, 4A and 4F) was inserted into PX459 V2.0 plasmid and transfected into U2OS, HEK293 and HAP1 cells with GenJet-OS (SignaGen), JetPRIME reagent (PolyPlus) and Turbofectin (Origene) respectively according to the manufacturer’s instructions. After 48 h of transfection, cells were subjected to transient selection with 2 μg/ml puromycin for 1–2 days to enrich for transfected cells. After puromycin selection, single colonies were grown for 2–4 weeks and then part of the colony was subjected to genomic DNA extraction with lysis buffer (10 mM Tris-HCl, pH 7.5, 10 mM of ethylene diamine tetra-acetic acid, 0.5% of sodium dodecyl sulfate, 10 mM of NaCl, 1 mg/mL of proteinase K) as previously described [85]. PCR screenings were done using p1F and p1R (p1F sequence: 5′-GAACACTAAATCAGGATGCTAATTCTAG-3′ and p1R sequence: 5′-TCTCTTTCTGCTAGAGCTTTAGCTC-3′) primers to assess for presence of insertions and deletions (InDels) that had been made in the region of interest. Any positive hits from the PCR screening were further verified by Sanger sequencing. In addition, part of the colony was harvested for western blotting and immunofluorescence analysis of localization and post-translational modification status of RanGAP1 as described in the previous sections. For cDNA sequencing, E3 mutants were subjected to RNA extraction with Trizol reagent (Invitrogen) and reverse transcription with SuperScript IV Reverse Transcriptase (Invitrogen). The cDNA was amplified by PCR using p2F and p2R (p2F sequence: 5′-CTACACATTTAAAACACCAGAAAAGG-3′ and p2R sequence: 5′-TTGAGCTTCCTGAACTTTTTGAAG-3′) primers and analyzed by Sanger sequencing.

### Dual luciferase reporter assay

For the *IL6* 3′UTR luciferase assay, U2OS and RanBP2-dE3 cells (4×10^4^ cells/well) were seeded in 24-well plates 16 h prior to transfection and transfected with psiCHECK2-Luc-IL6 (100 ng) or psiCHECK2-Luc-IL6-(Let7m) (100 ng) together with 50 nM has-Let7a-5p miRNA (MIMAT0000062, Dharmacon) or miRIDIAN microRNA Mimic Negative Control #1 (CN-001000-01-05, Dharmacon) using JetPRIME reagent (PolyPlus) according to the manufacturer’s instructions. Cells were collected 24 h after transfection and luciferase activities were measured with Dual-Luciferase Reporter Assay System (Promega) according to manufacturer’s instructions.

### In vivo sumoylation and ubiquitination assay

In vivo AGO1 sumoylation was analysed in unmodified and RanBP2-dE3 U2OS cells or unmodified and RanBP2-dE3-1 HEK293 cells as previously described [79,86,87]. Briefly, cells in 10 cm dishes were transfected with plasmids (2.5 μg each) expressing His6-SUMO1 or His6-SUMO2 and V5-Ubc9, together with FLAG/HA-tagged AGO1 (FH-AGO1) proteins (in pIRESneo) or empty plasmid negative control using JetPRIME reagent (PolyPlus) according to the manufacturer’s instructions. 24 h after transfection, the cells were harvested, and 10% were lysed in 2X SDS loading buffer (60mM Tris-HCl pH 6.8, 1% SDS, 100 mM DTT, 5% glycerol) to generate an input sample. 90% of the cells were resuspended in 0.2 mL lysis buffer containing 6 M Guanidinium-HCl, 100mM K_2_HPO_4_, 20mM Tris-HCl (pH 8.0), 100 mM NaCl, 0.1% Triton X-100, and 10 mM Imidazole, and incubated on ice 20 min. Lysates were passed through a 30G needle five times. Purification of the His6-SUMO1 or His6-SUMO2 conjugates was performed on 50 μL of Ni^2+^-NTA agarose beads (Qiagen) prewashed with lysis buffer, and incubated for 2–3 h at room temperature with end-over-end rotation. The beads were washed once with 1 mL of lysis buffer, and three times with 1 mL of wash buffer containing 8 M urea, 0.1 M Na_2_HPO_4_/NaH_2_PO_4_ (pH 6.4), 0.01 M Tris-HCl (pH 6.4), 10 mM Imidazole, 10 mM β-mercaptoethanol, and 0.1% Triton X-100 before elution in 2X SDS loading buffer.

To detect exogenous ubiquitin conjugates, cells were co-transfected with plasmids (2.5 μg each) expressing His-Myc-ubiquitin [78] and FH-AGO1 proteins or control plasmids and were treated with 10 μM MG132 for 7 h before harvesting. Ni^2+^-purified His6-ubiquitin (Ub) forms of FH-AGO1 followed by western blotting analysis were conducted as described above for the sumoylation assay.

To detect ubiquitin modifications on AGO1, cells were transfected with plasmids (2.5 μg each) expressing FH-AGO1 proteins or FH-EYFP (used as a negative control) and were treated with 10 μM MG132 for 7 h before harvesting. 24 h after transfection, cells were lysed in RIPA buffer (50 mM Tris-HCl, 150 mM NaCl, 1% Triton X-100, 1 mM EDTA, and complete protease inhibitor cocktail (Roche), pH 7.4) for immunoprecipitation. Cell lysates were incubated with Protein-G Sepharose beads coupled to the anti-HA antibody for 2–3 h at 4°C. The beads were washed three times with RIPA and then processed for immunoblotting.

### In vitro sumoylation assay

Assays for in vitro sumoylation of AGO1 mediated by RanBP2 were performed with the SUMOylation Assay Kit from Abcam (ab139470) according to the manufacturer’s protocol. Briefly, recombinant GST-tagged Human Ago1 protein (Abcam, ab162000) was incubated with active recombinant human RanBP2 protein (BP2△FG) (Abcam, ab268915) and other components supplied in the Kit. Reactions omitting ATP cofactors or with ATP but no BP2△FG, or with GST proteins as substrates were included as negative controls. Reactions were incubated at 30°C for 1.0 h and quenched by addition of 2X SDS-PAGE gel loading buffer followed by heating to 95°C for 5 minutes. Mixtures were then subjected to SDS-PAGE and immunoblot analyzed with anti-SUMO2/3 (1:1000 dilution, Abcam) or anti-GST antibodies (1:2000 dilution, Sino Biological) for detection of GST-AGO1 substrate proteins.

### Statistical analysis

Statistical tests were performed using two-tailed Student’s *t*-test to calculate P-values. P-value ≤ 0.05 was considered statistically significant. Results of statistical analysis are included in the corresponding figure legends.

### Ribosome fractionation

U2OS and RanBP2-dE3 cells were transfected with *IL6-li-HA* plasmid (15 μg) using GenJet-OS (SignaGen). 24 h post-transfection, cells were treated with 100 μg/mL cycloheximide (Sigma) for 15 minutes, harvested using trypsin and washed two times with PBS. Cells were then lysed in 1 mL lysis buffer containing 20 mM HEPES-KOH (pH 7.4), 5 mM MgCl_2_, 50 mM KCl, 1% Triton X-100, 100 μg/ml cycloheximide, complete protease inhibitor cocktail (Roche) and 1μL DEPC for 15 min. The lysates were cleared by centrifugation at 16,000*g* for 10 min at 4°C and 400 μL of the lysates were layered over a 20–50% sucrose gradient in polysome buffer (20 mM HEPES-KOH (pH 7.4), 5 mM MgCl_2_, 125 mM KCl, 100 μg/mL cycloheximide). The solution was centrifuged at 36,000 rpm for 2 h in a SW41Ti rotor (Beckman Coulter) at 4°C. Gradients were fractionated and absorbance at 254nm was measured using the Piston Gradient Fractionator (Biocomp) coupled with Bio-Rad EM1 UV monitor following the manufacture’s protocol.

RNA was isolated from each fraction using the Trizol reagent (Invitrogen) according to the manufacturer’s protocol. Fractions representing free RNA, monosome, light polysomes (2–4 ribosomes), medium polysomes (5–7 ribosomes) and heavy polysomes (>8 ribosomes) were identified using the gradient profiles generated and were pooled together. *In vitro* transcribed Renilla *luciferase* mRNA (a final amount of 0.3 ng after pooling) was spiked into the fractions before RNA extraction as an external control. Samples were processed for RT-qPCR.

### RT-qPCR

The RNA-containing samples were treated with DNase I (Invitrogen), reversed transcribed into cDNA with SuperScript IV Reverse Transcriptase (Invitrogen), analyzed by RT-qPCR (Applied Biosystems SYBR Green master mix) as per manufacture’s protocol. Primers used for RT-qPCR were, *α-tubulin* forward: 5′-CCAAGCTGGAGTTCTCTA-3′, reverse: 5′-AATCAGAGTGCTCCAGGG-3′; *IL6* mRNA forward: 5′-TGAGAGTAGTGAGGAACAAG-3′, reverse: 5′-CGCAGAATGAGATGAGTTG-3′; *IL6* intron forward: 5′-TAGCCCTGGAACTGCCAGCG-3′ (targets intron), reverse: 5′-ACTGGACCGAAGGCGCCTGT-3′ (spans intron and ORF); and Renilla *luciferase* forward: 5′-ATAACTGGTCCGCAGTGGTG-3′, reverse: 5′-TAAGAAGAGGCCGCGTTACC-3′. RT-qPCR data was analyzed using the ΔCT method where the relative expression of *IL6* and *tubulin* were normalized to that of Renilla *luciferase*.

### miRNA titration assay

U2OS and RanBP2-dE3 cells were seeded at a confluency of 20% in 6-well plates (0.3×10^6^ cells/well) and incubated for 24 h. The cells were then transfected with 48, 24, 12, or 6 pmol of miR-644 Mimic (Dharmacon) or miRIDIAN miR-144 Mimic (Dharmacon) as a negative control using JetPRIME reagent (PolyPlus) according to the manufacturer’s instructions. Cell lysates were collected 48 h after transfection and analyzed by immunoblotting.

### Cell fractionation

Cells were resuspended in Phi buffer containing 20 mM HEPES-KOH (pH7.4), 150 mM potassium acetate, 5 mM magnesium acetate, complete protease inhibitor cocktail (Roche) and 10 mM PMSF. Cells were lysed in Phi buffer with 0.5% Triton X-100 and 0.25% sodium deoxycholate for 1 min and spun at 1000*g* for 5 min at 4°C. The supernatant was kept as the cytoplasmic/ER fraction and was spun at 15,000*g* for 15 min to clear. Pelleted nuclei were washed two times to remove any unlysed cells.

### RNA-immunoprecipitation

U2OS cells or unmodified and RanBP2-E3ins HAP1 cells were transfected with *IL6-Δi-HA* or *IL6-1i-HA* plasmids (16 μg for U2OS and 12 μg for HAP1) using JetPrime reagent (PolyPlus) or Turbofectin (OriGene). 24-48h post-transfection, cell fractionation was performed as described above with the presence of SUPERase-IN RNase inhibitior (Thermo Fisher). The pelleted nuclei were lysed in high salt buffer (Phi buffer with 1% Triton X-100 and 145mM NaCl) for 30 min with rotation at 4°C. IP buffer (Phi buffer with 1% Triton X-100) was then added to dilute out the salt. The fractions were then treated with DNase I (Thermo Fisher) at room temperature for 20 min and were spun at 10,000*g* for 10 min to clear. 5% of the cleared cytoplasmic/ER and nuclear fractions were collected and processed for immunoblotting or RNA extraction, respectively. For the remaining, the fractions were split equally into two, mixed with 10 μg of control (C1) or anti-AGO synthetic antibodies [59] and incubated overnight with rotation at 4°C. Flag M2 beads (Sigma A2220) were blocked with 1% BSA, washed 3 times with IP buffer and incubated with the lysate-synthetic antibody mixture for 3 hours with rotation at 4°C. The beads were washed 5 times in IP buffer. 10% of the beads were collected and processed for immunoblotting. RNA was collected from the remaining beads with Trizol Reagent (Invitrogen) according to the manufacturer’s protocol and processed for RT-qPCR analysis as described above.

## Supporting information

S1 Fig Most ANE1-associated cytokine genes are not depleted of adenines and have low 5IMP scores. (A) For each gene in the human genome, the longest tract of adenine-less sequence in the first 99 nucleotides of the open reading frame was tabulated as in Palazzo *et al*, 2007, and plotted, with the *x-axis* representing the length of these tracts, and the *y-axis* representing the fraction of genes in each set with these tract lengths. This was tabulated for all genes that contain an SSCR that lacks introns in their 5′UTR (“SSCR 5UI-”; blue), which are known to be positively regulated by RanBP2 [1], and for genes that lacked SSCRs (“Non-SSCR”; green). To control for the length of adenine-less tracts in random human DNA sequences, the frequency of adenine-less tract length was also tabulated for regions 3 kb upstream (yellow) and 3 kb downstream (red) of protein coding genes. The adenine-less tract lengths for ANE1-associated cytokine genes (see S1 Table) are labeled. (B) For each gene in the human genome the 5IMP score was calculated, as described in Cenik et al., 2017, and plotted with the *x-axis* representing binned 5IMP scores, and the *y-axis* representing the fraction of genes in each set with these scores. This was tabulated for all genes that contain an SSCR that lacks introns in their 5′UTR (“SSCR 5UI-”; blue), for genes that contain both an SSCR and one or more introns in their 5′UTR (“SSCR 5UI+”; red) and for all genes that contain one or more introns in their 5′UTR (“All Genes 5UI+”; green). The 5IMP scores for ANE1-associated cytokine genes are labeled.
(TIF)

S2 Fig RanBP2 represses the expression of IL6 independently of splicing. (A) Schematic of the *IL6* constructs tested. This includes an intronless version of *IL6* (*IL6-Δi*), a version containing the first endogenous *IL6* intron (*IL6-1i*) or the *ftz* intron (*IL6-1f*) both inserted at the endogenous first exon-exon boundary. (B-C) U2OS cells were infected with lentivirus that delivered shRNA1 against RanBP2 or control virus. Three days post-infection, cells were transfected with plasmids containing the indicated reporter genes. 18–24 h post-transfection cell lysates were collected and separated by SDS-PAGE. The level of each protein was analyzed by immunoblot for HA, and α-tubulin as a loading control (B). The levels of each HA-tagged protein and α-tubulin were quantified using densitometry analysis (C). The HA/tubulin ratio was normalized to *IL6-Δi* transfected control shRNA-treated cells and plotted with each bar being the average of three independent experiments ± SEM. *P = 0.01–0.05 (Student’s *t*-test).
(TIF)

S3 Fig Schematic of mutant RanBP2 proteins from CRISPR/Cas9-engineered cell lines. (A) Schematic of the domains encoded by a portion of the human *RanBP2* gene, including the end of exon 20, all of exons 21 through 24, and the beginning of exon 25. SIM: SUMO interacting motif, IR: internal repeat. The amino acids that are denoted by asterisks have been shown to be required for SUMO E3-ligase activity [2]. (B-E) Schematics of the mutant RanBP2 proteins encoded by mRNAs derived from the various mutant cell lines. PTC: premature termination codon.
(TIF)

S4 Fig Localization of RanBP2 variants. (A) Unmodified and RanBP2-dE3 U2OS cells were fixed and immunostained for RanBP2 and DAPI stained to visualize DNA. Note that the modified RanBP2-dE3 proteins localize to the nuclear rim like the unmodified protein. (B) RanBP2-dE3 cells that stably express a GFP-RanBP2 with three ANE1 mutations were fixed and immunostained for GFP (this was done as the expression of this construct is too low to detect by GFP fluorescence alone) and DAPI stained to visualize DNA. Scale bar = 10 μm.
(TIF)

S5 Fig Genomic analysis and sequencing of mutant RanBP2 genes from CRISPR/Cas9-engineered cell lines. (A) Genomic DNA was isolated from unmodified and mutant RanBP2-E3ins HAP1 cells and amplified with p1F and p1R primers (see Fig 2A). The amplified fragment from RanBP2-E3ins HAP1 cells was sequenced and compared to exon 21 of the human *RanBP2* gene (B). Note that the PAM site for the guide RNA (gRNA-dE3-1#, see Fig 2B) is indicated. (C) Genomic DNA was isolated from unmodified and mutant RanBP2-dE3-1 HEK293 cells and amplified with p1F and p1R primers. The two alleles (f1 and f2) were sequenced and compared to exon 21 of the human *RanBP2* gene (D). Note the PAM sites for gRNA-dE3-1# and the position of a pre-mature stop codon in f2 are indicated. (E) Genomic DNA was isolated from unmodified and mutant RanBP2-dE3-2 HEK293 cells and amplified with p1F and p1R primers. The two alleles (f1 and f2) were sequenced and compared to exon 21 and intron 21 of the human *RanBP2* gene (F). Note the PAM sites for gRNA-dE3-3# are indicated.
(TIF)

S6 Fig Identification of RanBP2-responsive elements in the 5′ and 3′UTR of the human *IL6* mRNA. **(A-C) Testing the role of the SSCR in the regulation of IL6 by RanBP2.** (A) Schematic of the original *IL6* construct (*IL6-Δi*) and a version where the endogenous SSCR was replaced with the mouse *MHC* SSCR derived from the *h2kb* gene (*MHC-IL6-Δi*). (B-C) U2OS cells were infected with lentivirus that delivered shRNA1 against RanBP2 or control virus. Three days post-infection, cells were transfected with plasmids containing the indicated reporter genes. 18–24 h post-transfection cell lysates were collected and separated by SDS-PAGE. The level of each protein was analyzed by immunoblot for HA, and α-tubulin as a loading control (B). The levels of each HA-tagged protein and α-tubulin were quantified using densitometry analysis. The HA/tubulin ratio was normalized to *IL6-Δi* transfected control shRNA-treated cells and plotted (C) with each bar being the average of three independent experiments ± SEM. *P = 0.01–0.05 (Student’s *t*-test). **(D-H) Testing the roles of the 5′ and 3′UTRs in the regulation of IL6 by RanBP2.** (D) Schematic of the various intron-containing *IL6-HA* constructs (*IL6-1i*), where the 5′UTR was replaced with that of the *ftz* reporter (*5F-IL6-1i*) or the β-globin reporter (*5βG-IL6-1i*), and the 3′UTR was replaced with that of the ftz reporter (*IL6-1i-3F*). (E-H) Expression of the reporters was performed as in (B) and quantified as in (C), with each bar being the average of three independent experiments ± SEM. *P = 0.01–0.05, n.s. indicates no significant difference (Student’s *t*-test). **(I-K) Dissecting the *IL6* 3′UTRs to determine the RanBP2-regulatory element.** (I) Schematic of the *IL6-1i*, *IL6-1i-3del1*, and *IL6-1i-3del2* constructs. 3del1 consists of the deletion of first 110 nucleotides of the *IL6* 3′UTR whereas 3del2 consists of the deletion of 111–439 nucleotides of the *IL6* 3′UTR. (J-K) Expression of the reporters was performed as in (B) and quantified as in (C), with each bar being the average of three independent experiments ± SEM. *P = 0.01–0.05, n.s. indicates no significant difference (Student’s *t*-test).
(TIF)

S7 Fig RanBP2 is required for elevated levels of AGO1 but not AGO2 in U2OS cells. (A) U2OS cells were infected with lentivirus that delivered shRNA1 against RanBP2 or control virus. Three days post-infection, cells were lysed, separated by SDS-PAGE, and immunoblotted with antibodies against AGO1, AGO2, RanBP2, RanGAP1, and α-tubulin. (B) Unmodified U2OS, RanBP2-dE3 and RanBP2-dE3 cells which stably express GFP-RanBP2 with three ANE1 mutations (“ANE1-GFP”) were lysed, separated by SDS-PAGE, and immunoblotted with antibodies against AGO1, AGO2, RanBP2, RanGAP1, and α-tubulin.
(TIF)

S8 Fig RanBP2 stability does not require Ubc9. U2OS cells were infected with lentivirus that delivered a mixture of two shRNAs against Ubc9 or control virus. Five days post-infection, cells were lysed, separated by SDS-PAGE, and immunoblotted with antibodies against RanBP2, Ubc9, and α-tubulin.
(TIF)

S9 Fig RanBP2 stabilizes overexpressed FH-AGO1. Unmodified U2OS, and RanBP2-dE3 cells were co-transfected with *FH-AGO1* and *H1B-GFP*. 18 h post-transfection cells were treated with cycloheximide (CHX, 100 μM) in the presence of MG132 (10 μM) or DMSO for 7 hr. Cell lysates were collected, separated by SDS-PAGE, and immunoblotted with antibodies against HA, GFP, RanBP2 and α-tubulin (A). FH-AGO1 and H1B-GFP protein levels were quantified using densitometry analysis and the ratio of FH-AGO1/H1B-GFP was normalized to DMSO-treated unmodified U2OS cells (B). Each bar is the average of three independent experiments ± SEM.*P = 0.01–0.05, **P = 0.001–0.01, n.s. indicates no significant difference (Student’s *t*-test).
(TIF)

S10 Fig Densitometry quantification of AGO1 sumoylation and ubiquitination. (A) Densitometry signals for isolated His6-SUMO2-FH-AGO1 (see Fig 7A) were quantified. The signal was normalized to unmodified (WT) cells and plotted. (B) Densitometry signals for isolated His6-SUMO1-FH-AGO1 (see Fig 7B) were quantified and plotted as in (A). (C) Densitometry signals for isolated His6-SUMO2-FH-AGO1 (see Fig 7C) were quantified and plotted as in (A). (D) Densitometry signals for isolated His-Myc-Ub-FH-AGO1 (see Fig 7E) were quantified and plotted as in (A). (E) Densitometry signals for isolated Ub(n)-FH-AGO1 (see Fig 7F) were quantified and plotted as in (A). (F) Densitometry signals for isolated Ub(n)-FH-AGO1 (see Fig 7G) were quantified and plotted as in (A). Each bar is the average of three independent experiments ± SEM.*P = 0.01–0.05, **P = 0.001–0.01, ***P < 0.001, n.s. indicates no significant difference (Student’s *t*-test).
(TIF)

S11 Fig The K400R mutant of AGO1 is defective in IL6 silencing and does not require RanBP2-dependent sumoylation for its stability. (A) Densitometry signals for IL6-HA and H1B-GFP in RanBP2-dE3 U2OS cells expressing FH-AGO1 and FH-AGO1^K400R^ (see Fig 7I) were quantified. The signal was normalized to cells expressing FH-AGO1. Each bar is the average of three independent experiments ± SEM. **P = 0.001–0.01 (Student’s *t*-test). (B-C) Unmodified and RanBP2-dE3 U2OS cells were transfected with FH-AGO1 and FH-AGO1^K400R^ and 18 h post-transfection treated with cycloheximide (CHX) for the indicated times to determine the decay rates of the two forms of FH-AGO1. Cell lysates were collected, separated by SDS-PAGE, and immunoblotted with antibodies against HA, GFP, Tubulin and RanGAP1. Note that FH-AGO1 primarily migrates just below the 100 kDa marker, while the FH-AGO1^K400R^ migrates between the 100 and 135 kDa markers (in agreement with Fig 7I). The levels of the HA and α-tubulin immunoblot signals were analyzed by densitometry analysis and the ratio of FH-AGO1/α-tubulin was normalized to the zero time point. Each point is the average of three independent experiments ± SEM.*P = 0.01–0.05 (Student’s *t*-test).
(TIF)

S12 Fig The majority of AGO1 and AGO2 is nuclear in HAP1 and HEK293 cells. Total, Cytoplasmic/ER and nuclear fractions were isolated from unmodified and RanBP2-E3ins HAP1 cells (A) or HEK293 cells (B), separated by SDS-PAGE and immunoprobed for AGO1, AGO2, α-tubulin (cytosolic marker), Trapα (ER marker) and Aly (nuclear marker). Note that in all cell lines, the majority of the Argonaute proteins are nuclear.
(TIF)

S13 Fig A substantial fraction of overexpressed FH-AGO1 is cytoplasmic. (A-B) U2OS cells were transfected with FH-AGO1 and after allowing expression for 18 hrs were fixed and immunostained for FLAG and DAPI stained to visualize DNA. Scale bar = 10 μm. A representative cell is shown (A) and the percent of the total integrated fluorescence in the cytoplasm and nucleus were quantified, each bar representing the average and standard error for 74 cells from two independent experiments. (C) Total, cytoplasmic/ER and nuclear fractions were isolated from U2OS cells that expressed FH-AGO1. Proteins from the lysates were separated by SDS-PAGE and immunoprobed for HA, α-tubulin (cytosolic marker), Trapα (ER marker) and Aly (nuclear marker).
(TIF)

S1 Table ANE1-associated cytokines from case reports dating back to 1998. (DOCX)

S1 Text Most ANE1-associated cytokine genes have SSCRs that contain adenines and have low 5IMP scores. RanBP2-mediated translation inhibition of the IL6-HA reporter requires cis-elements in both the IL6 5′UTR and 3′UTRs.
(DOC)
