# Supplementary figures and images for: RanBP2/Nup358 enhances miRNA activity by sumoylating Argonautes
Source: PLoS Genet. 2021 Feb 18;17(2):e1009378. doi: 10.1371/journal.pgen.1009378 (PMC7924746; doi:10.1371/journal.pgen.1009378)

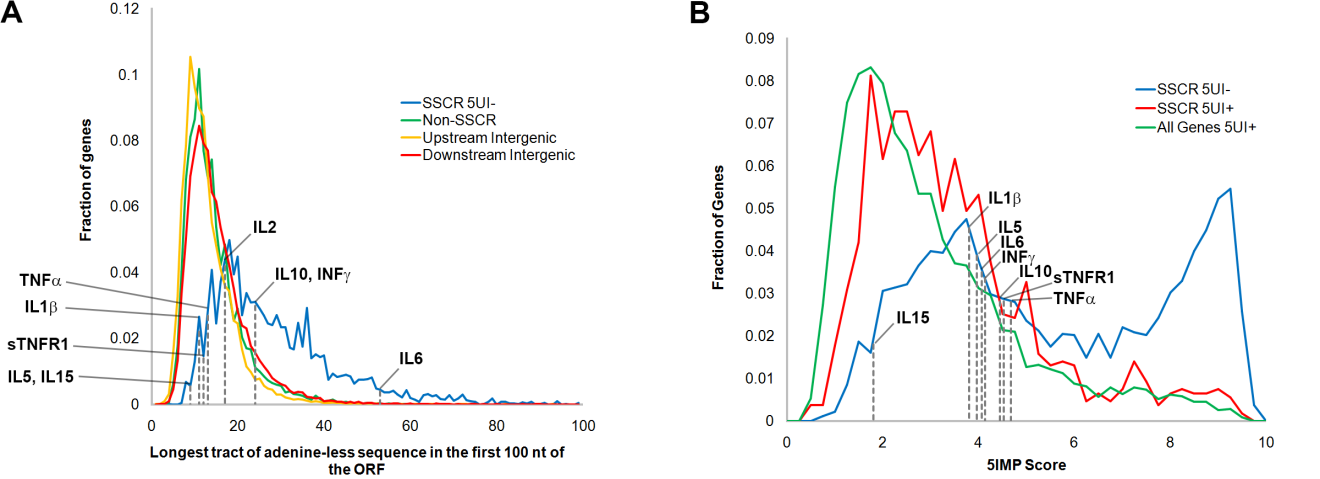

Supplement: S1 Fig — (A) For each gene in the human genome, the longest tract of adenine-less sequence in the first 99 nucleotides of the open reading frame was tabulated as in Palazzo et al, 2007, and plotted, with the x-axis representing the length of these tracts, and the y-axis representing the fraction of genes in each set with these tract lengths. This was tabulated for all genes that contain an SSCR that lacks introns in their 5′UTR (“SSCR 5UI-”; blue), which are known to be positively regulated by RanBP2 [1], and for genes that lacked SSCRs (“Non-SSCR”; green). To control for the length of adenine-less tracts in random human DNA sequences, the frequency of adenine-less tract length was also tabulated for regions 3 kb upstream (yellow) and 3 kb downstream (red) of protein coding genes. The adenine-less tract lengths for ANE1-associated cytokine genes (see S1 Table) are labeled. (B) For each gene in the human genome the 5IMP score was calculated, as described in Cenik et al., 2017, and plotted with the x-axis representing binned 5IMP scores, and the y-axis representing the fraction of genes in each set with these scores. This was tabulated for all genes that contain an SSCR that lacks introns in their 5′UTR (“SSCR 5UI-”; blue), for genes that contain both an SSCR and one or more introns in their 5′UTR (“SSCR 5UI+”; red) and for all genes that contain one or more introns in their 5′UTR (“All Genes 5UI+”; green). The 5IMP scores for ANE1-associated cytokine genes are labeled. (TIF) [file pgen.1009378.s001.tif]

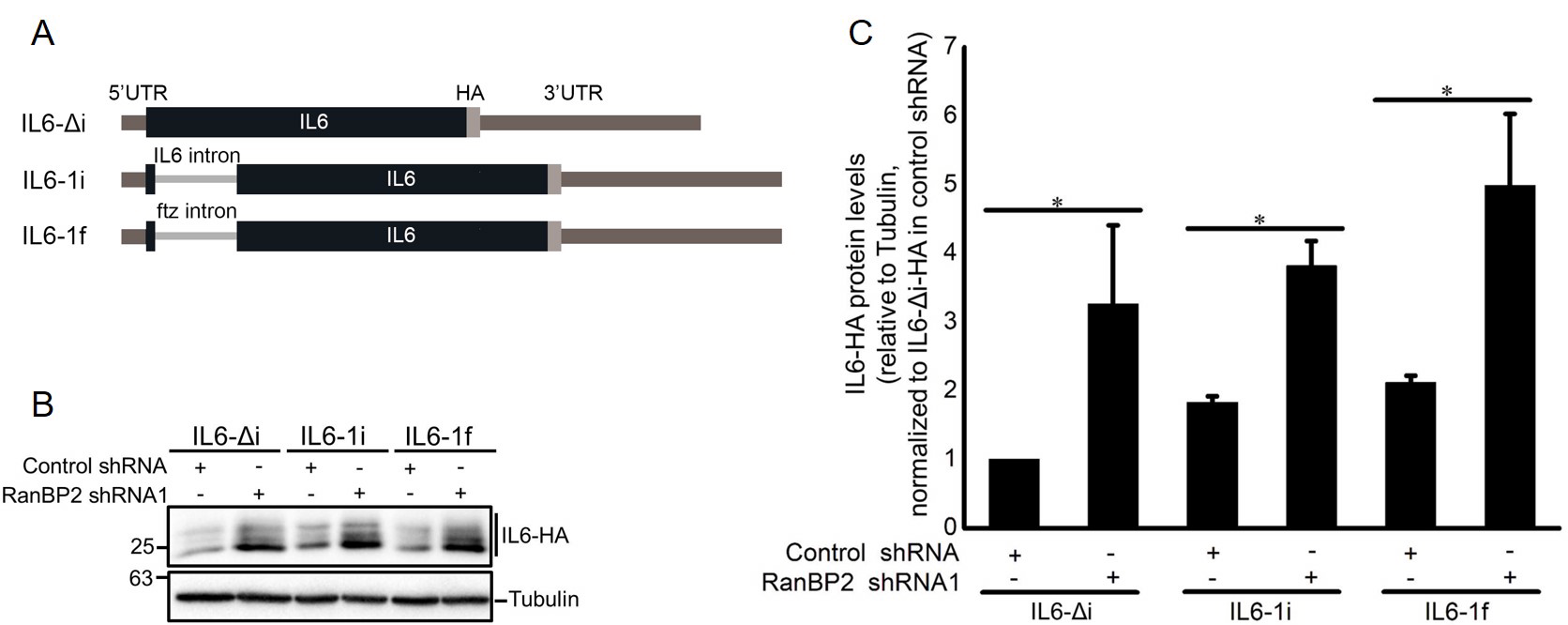

Supplement: S2 Fig — (A) Schematic of the IL6 constructs tested. This includes an intronless version of IL6 (IL6-Δi), a version containing the first endogenous IL6 intron (IL6-1i) or the ftz intron (IL6-1f) both inserted at the endogenous first exon-exon boundary. (B-C) U2OS cells were infected with lentivirus that delivered shRNA1 against RanBP2 or control virus. Three days post-infection, cells were transfected with plasmids containing the indicated reporter genes. 18–24 h post-transfection cell lysates were collected and separated by SDS-PAGE. The level of each protein was analyzed by immunoblot for HA, and α-tubulin as a loading control (B). The levels of each HA-tagged protein and α-tubulin were quantified using densitometry analysis (C). The HA/tubulin ratio was normalized to IL6-Δi transfected control shRNA-treated cells and plotted with each bar being the average of three independent experiments ± SEM. *P = 0.01–0.05 (Student’s t-test). (TIF) [file pgen.1009378.s002.tif]

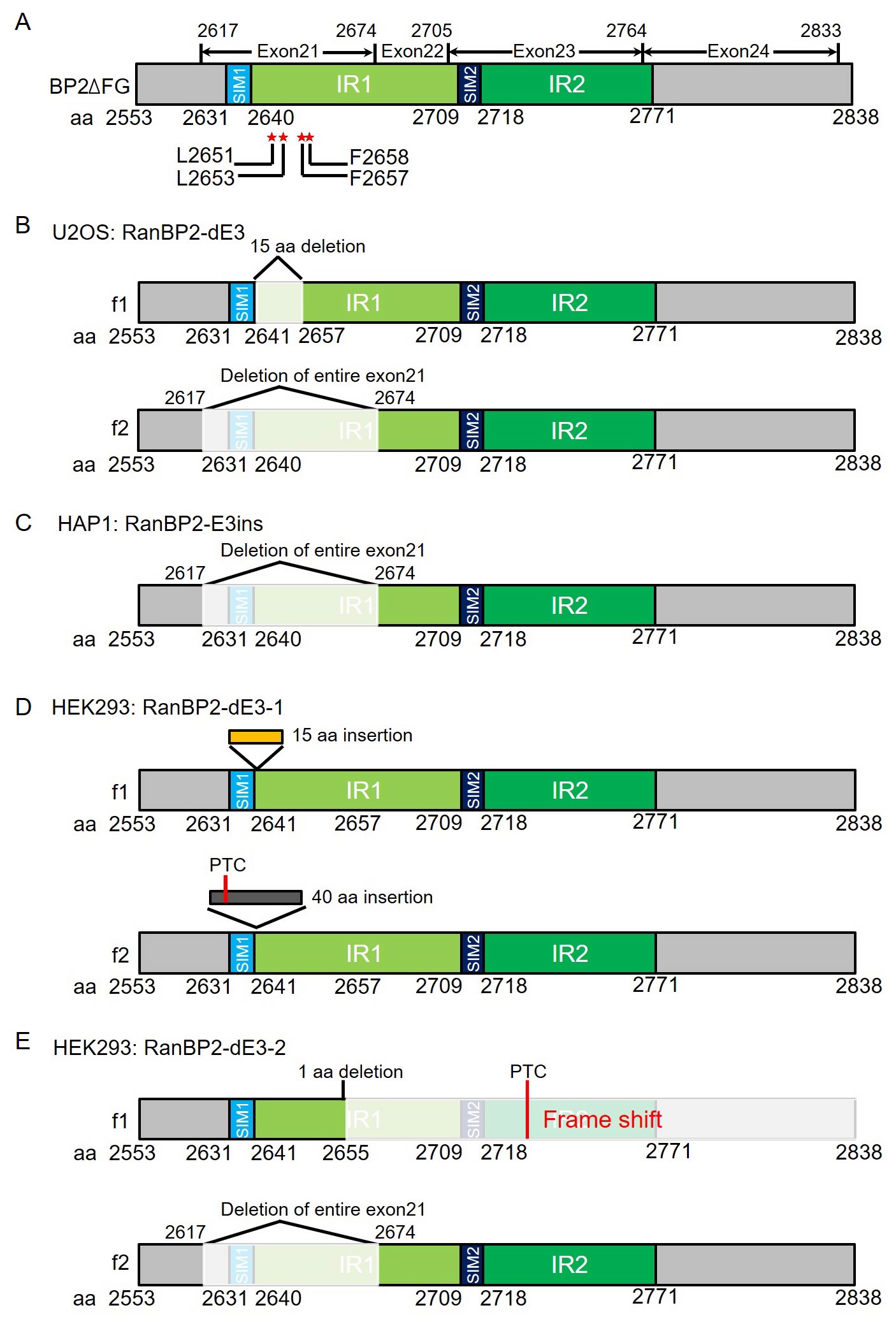

Supplement: S3 Fig — (A) Schematic of the domains encoded by a portion of the human RanBP2 gene, including the end of exon 20, all of exons 21 through 24, and the beginning of exon 25. SIM: SUMO interacting motif, IR: internal repeat. The amino acids that are denoted by asterisks have been shown to be required for SUMO E3-ligase activity [2]. (B-E) Schematics of the mutant RanBP2 proteins encoded by mRNAs derived from the various mutant cell lines. PTC: premature termination codon. (TIF) [file pgen.1009378.s003.tif]

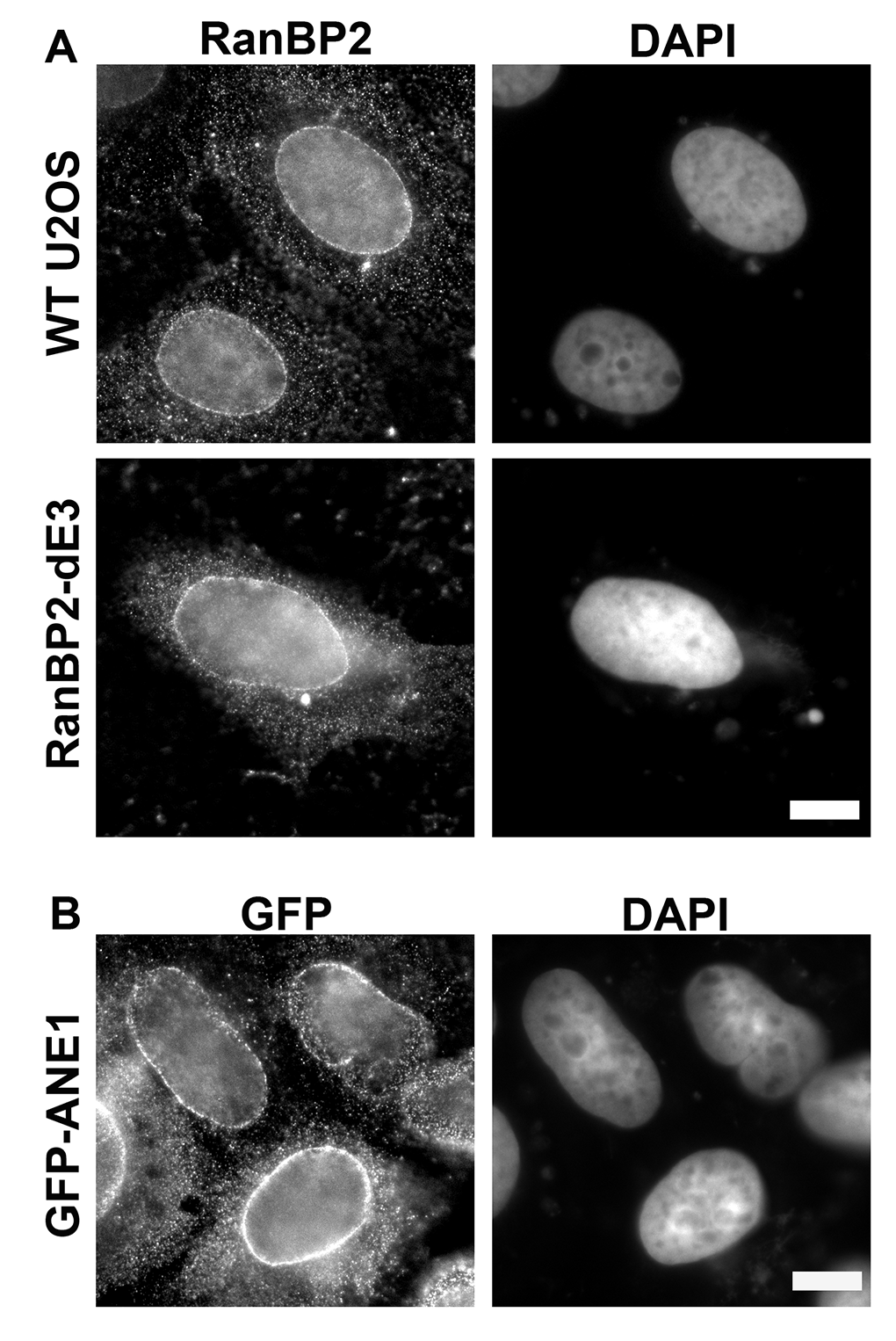

Supplement: S4 Fig — (A) Unmodified and RanBP2-dE3 U2OS cells were fixed and immunostained for RanBP2 and DAPI stained to visualize DNA. Note that the modified RanBP2-dE3 proteins localize to the nuclear rim like the unmodified protein. (B) RanBP2-dE3 cells that stably express a GFP-RanBP2 with three ANE1 mutations were fixed and immunostained for GFP (this was done as the expression of this construct is too low to detect by GFP fluorescence alone) and DAPI stained to visualize DNA. Scale bar = 10 μm. (TIF) [file pgen.1009378.s004.tif]

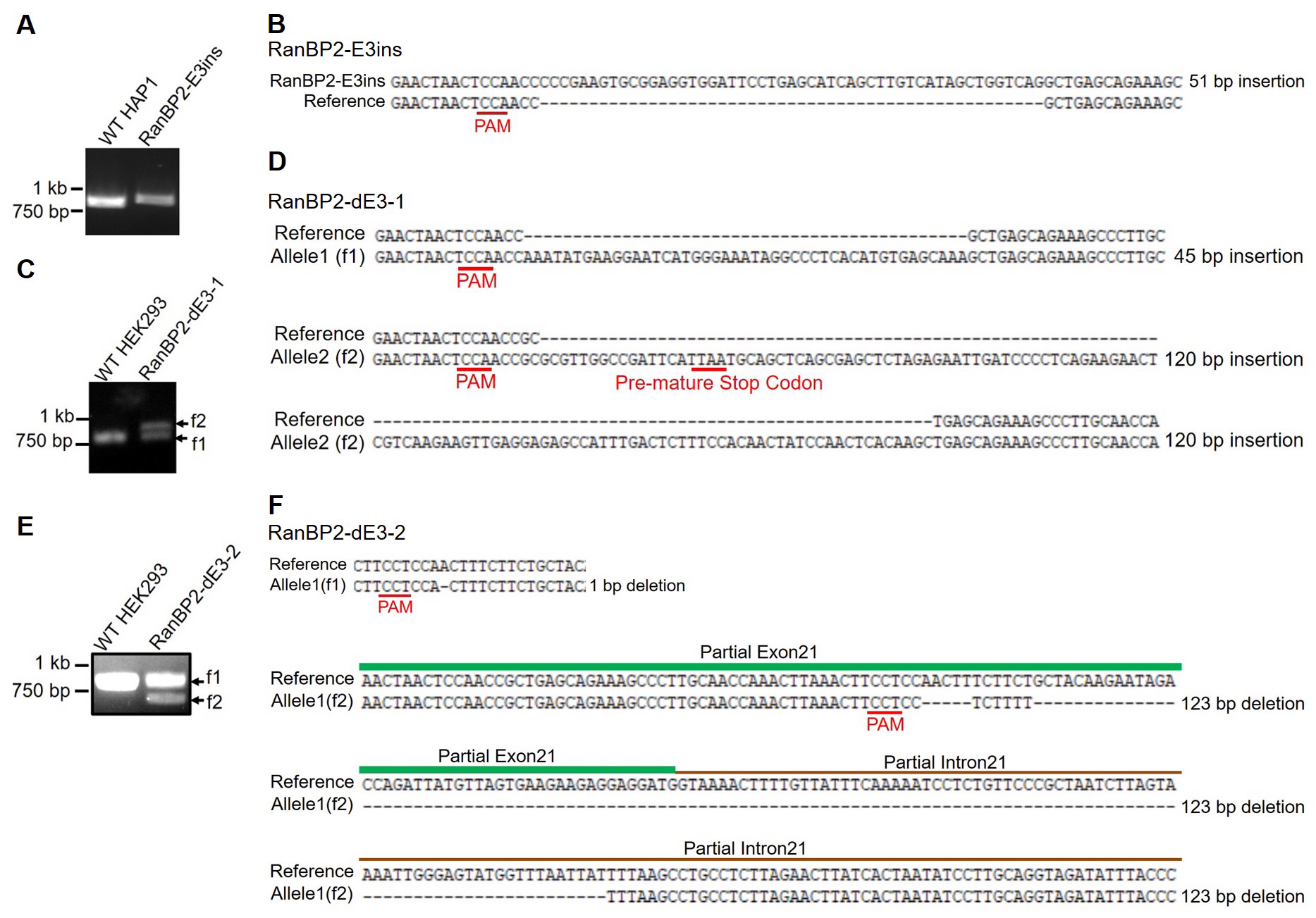

Supplement: S5 Fig — (A) Genomic DNA was isolated from unmodified and mutant RanBP2-E3ins HAP1 cells and amplified with p1F and p1R primers (see Fig 2A). The amplified fragment from RanBP2-E3ins HAP1 cells was sequenced and compared to exon 21 of the human RanBP2 gene (B). Note that the PAM site for the guide RNA (gRNA-dE3-1#, see Fig 2B) is indicated. (C) Genomic DNA was isolated from unmodified and mutant RanBP2-dE3-1 HEK293 cells and amplified with p1F and p1R primers. The two alleles (f1 and f2) were sequenced and compared to exon 21 of the human RanBP2 gene (D). Note the PAM sites for gRNA-dE3-1# and the position of a pre-mature stop codon in f2 are indicated. (E) Genomic DNA was isolated from unmodified and mutant RanBP2-dE3-2 HEK293 cells and amplified with p1F and p1R primers. The two alleles (f1 and f2) were sequenced and compared to exon 21 and intron 21 of the human RanBP2 gene (F). Note the PAM sites for gRNA-dE3-3# are indicated. (TIF) [file pgen.1009378.s005.tif]

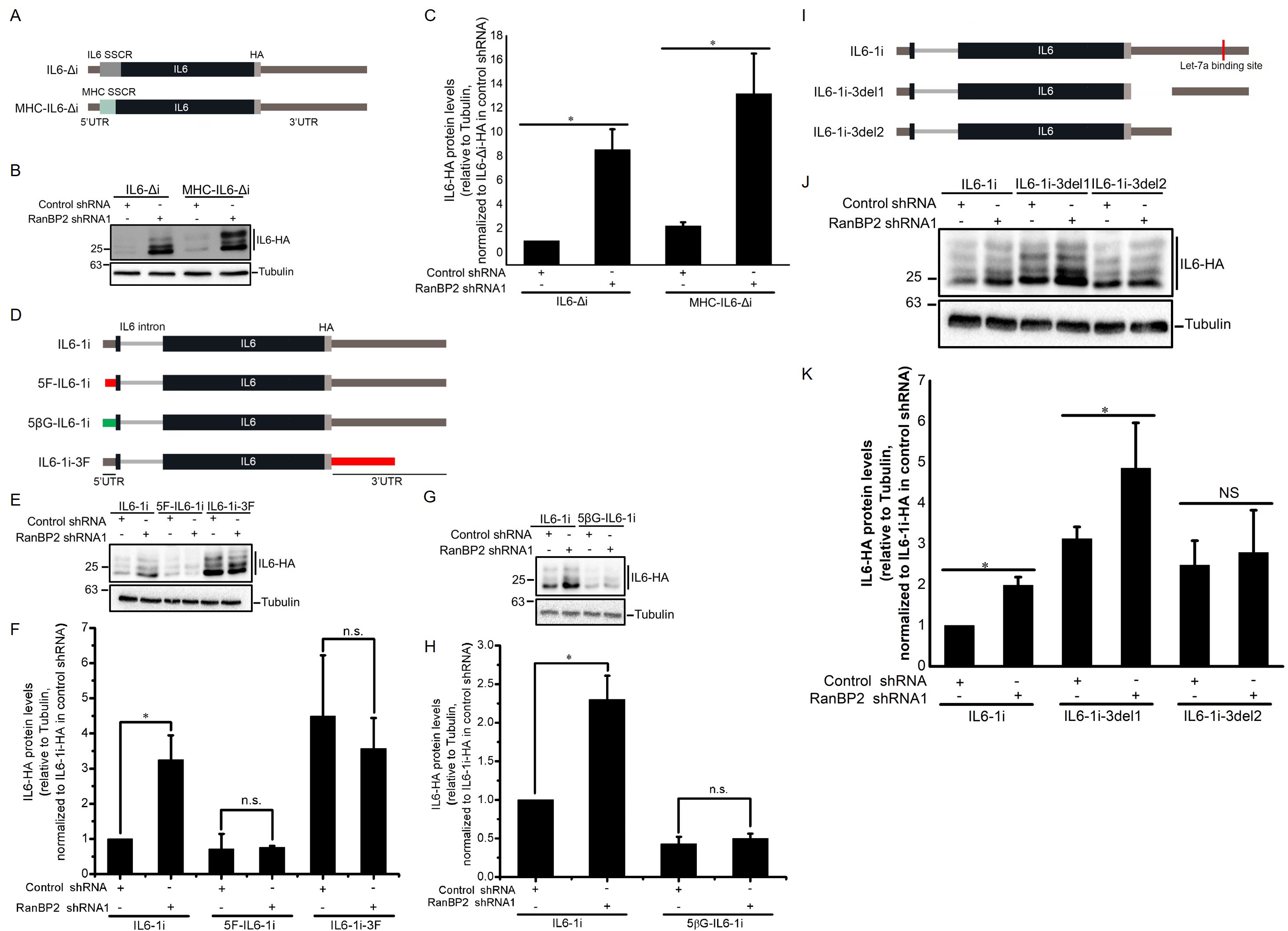

Supplement: S6 Fig — (A-C) Testing the role of the SSCR in the regulation of IL6 by RanBP2. (A) Schematic of the original IL6 construct (IL6-Δi) and a version where the endogenous SSCR was replaced with the mouse MHC SSCR derived from the h2kb gene (MHC-IL6-Δi). (B-C) U2OS cells were infected with lentivirus that delivered shRNA1 against RanBP2 or control virus. Three days post-infection, cells were transfected with plasmids containing the indicated reporter genes. 18–24 h post-transfection cell lysates were collected and separated by SDS-PAGE. The level of each protein was analyzed by immunoblot for HA, and α-tubulin as a loading control (B). The levels of each HA-tagged protein and α-tubulin were quantified using densitometry analysis. The HA/tubulin ratio was normalized to IL6-Δi transfected control shRNA-treated cells and plotted (C) with each bar being the average of three independent experiments ± SEM. *P = 0.01–0.05 (Student’s t-test). (D-H) Testing the roles of the 5′ and 3′UTRs in the regulation of IL6 by RanBP2. (D) Schematic of the various intron-containing IL6-HA constructs (IL6-1i), where the 5′UTR was replaced with that of the ftz reporter (5F-IL6-1i) or the β-globin reporter (5βG-IL6-1i), and the 3′UTR was replaced with that of the ftz reporter (IL6-1i-3F). (E-H) Expression of the reporters was performed as in (B) and quantified as in (C), with each bar being the average of three independent experiments ± SEM. *P = 0.01–0.05, n.s. indicates no significant difference (Student’s t-test). (I-K) Dissecting the IL6 3′UTRs to determine the RanBP2-regulatory element. (I) Schematic of the IL6-1i, IL6-1i-3del1, and IL6-1i-3del2 constructs. 3del1 consists of the deletion of first 110 nucleotides of the IL6 3′UTR whereas 3del2 consists of the deletion of 111–439 nucleotides of the IL6 3′UTR. (J-K) Expression of the reporters was performed as in (B) and quantified as in (C), with each bar being the average of three independent experiments ± SEM. *P = 0.01–0.05, n.s. indicates no sign [file pgen.1009378.s006.tif]

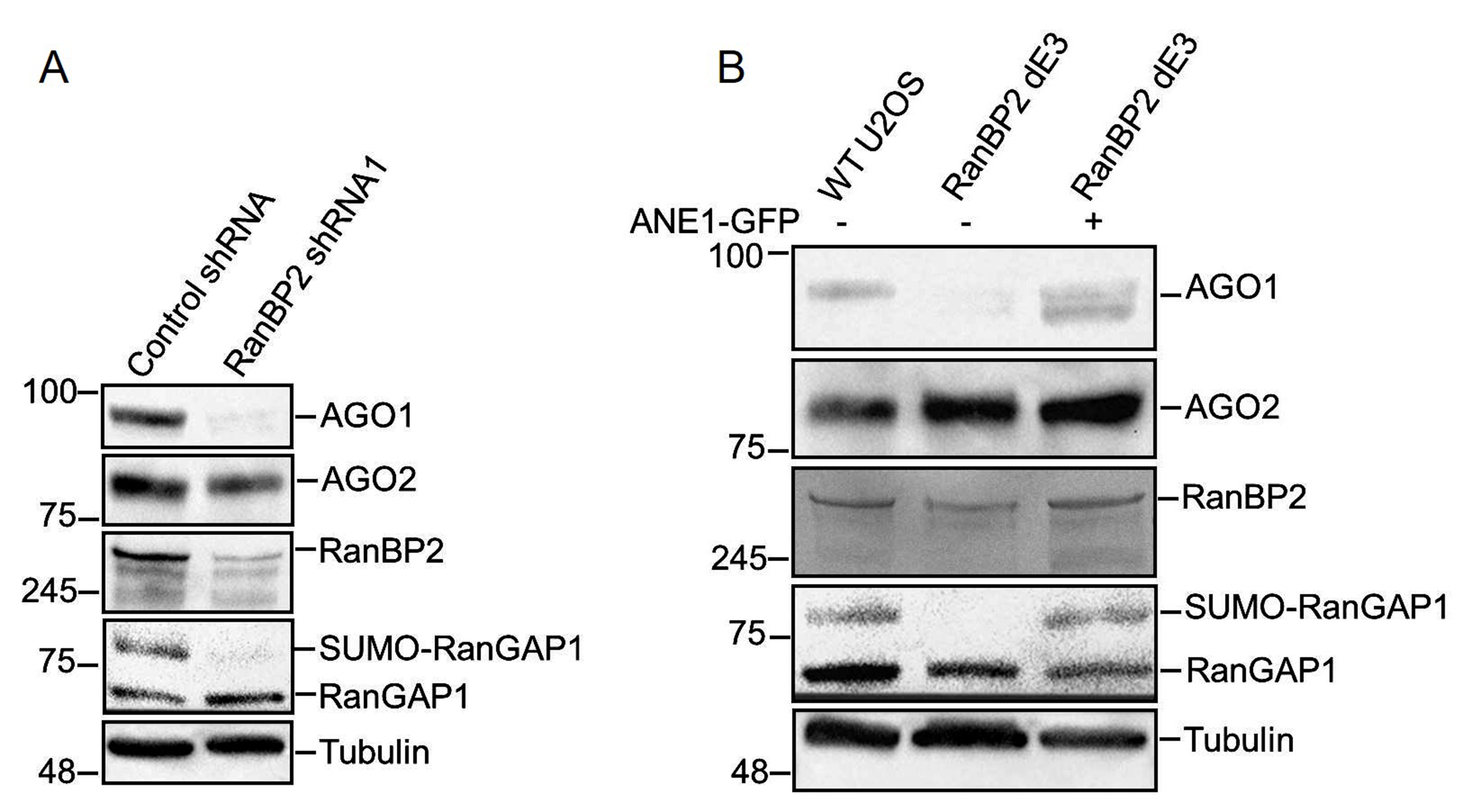

Supplement: S7 Fig — (A) U2OS cells were infected with lentivirus that delivered shRNA1 against RanBP2 or control virus. Three days post-infection, cells were lysed, separated by SDS-PAGE, and immunoblotted with antibodies against AGO1, AGO2, RanBP2, RanGAP1, and α-tubulin. (B) Unmodified U2OS, RanBP2-dE3 and RanBP2-dE3 cells which stably express GFP-RanBP2 with three ANE1 mutations (“ANE1-GFP”) were lysed, separated by SDS-PAGE, and immunoblotted with antibodies against AGO1, AGO2, RanBP2, RanGAP1, and α-tubulin. (TIF) [file pgen.1009378.s007.tif]

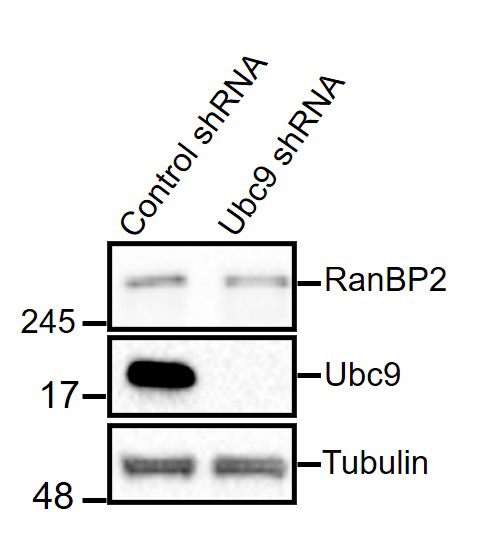

Supplement: S8 Fig — U2OS cells were infected with lentivirus that delivered a mixture of two shRNAs against Ubc9 or control virus. Five days post-infection, cells were lysed, separated by SDS-PAGE, and immunoblotted with antibodies against RanBP2, Ubc9, and α-tubulin. (TIF) [file pgen.1009378.s008.tif]

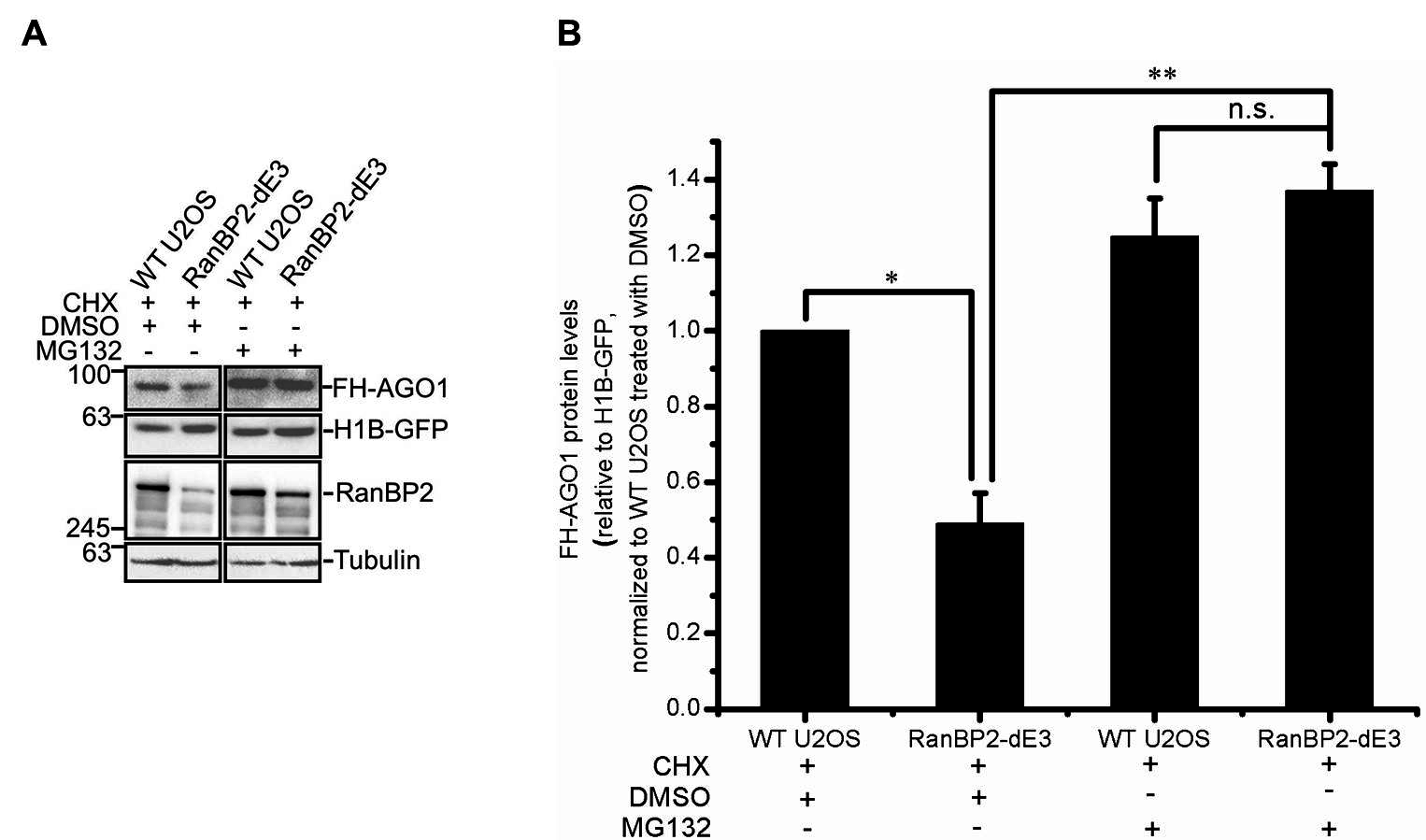

Supplement: S9 Fig — Unmodified U2OS, and RanBP2-dE3 cells were co-transfected with FH-AGO1 and H1B-GFP. 18 h post-transfection cells were treated with cycloheximide (CHX, 100 μM) in the presence of MG132 (10 μM) or DMSO for 7 hr. Cell lysates were collected, separated by SDS-PAGE, and immunoblotted with antibodies against HA, GFP, RanBP2 and α-tubulin (A). FH-AGO1 and H1B-GFP protein levels were quantified using densitometry analysis and the ratio of FH-AGO1/H1B-GFP was normalized to DMSO-treated unmodified U2OS cells (B). Each bar is the average of three independent experiments ± SEM.*P = 0.01–0.05, **P = 0.001–0.01, n.s. indicates no significant difference (Student’s t-test). (TIF) [file pgen.1009378.s009.tif]

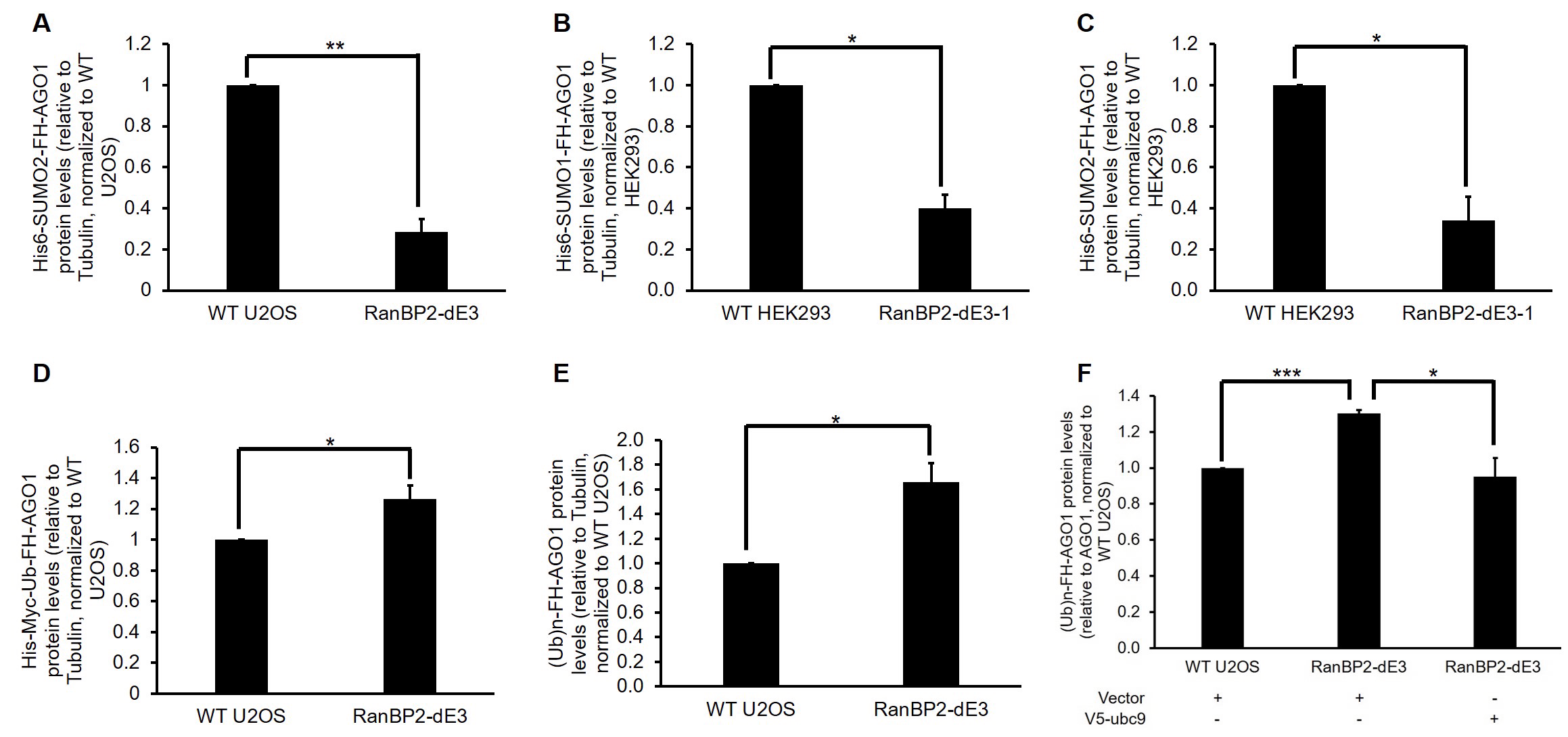

Supplement: S10 Fig — (A) Densitometry signals for isolated His6-SUMO2-FH-AGO1 (see Fig 7A) were quantified. The signal was normalized to unmodified (WT) cells and plotted. (B) Densitometry signals for isolated His6-SUMO1-FH-AGO1 (see Fig 7B) were quantified and plotted as in (A). (C) Densitometry signals for isolated His6-SUMO2-FH-AGO1 (see Fig 7C) were quantified and plotted as in (A). (D) Densitometry signals for isolated His-Myc-Ub-FH-AGO1 (see Fig 7E) were quantified and plotted as in (A). (E) Densitometry signals for isolated Ub(n)-FH-AGO1 (see Fig 7F) were quantified and plotted as in (A). (F) Densitometry signals for isolated Ub(n)-FH-AGO1 (see Fig 7G) were quantified and plotted as in (A). Each bar is the average of three independent experiments ± SEM.*P = 0.01–0.05, **P = 0.001–0.01, ***P < 0.001, n.s. indicates no significant difference (Student’s t-test). (TIF) [file pgen.1009378.s010.tif]

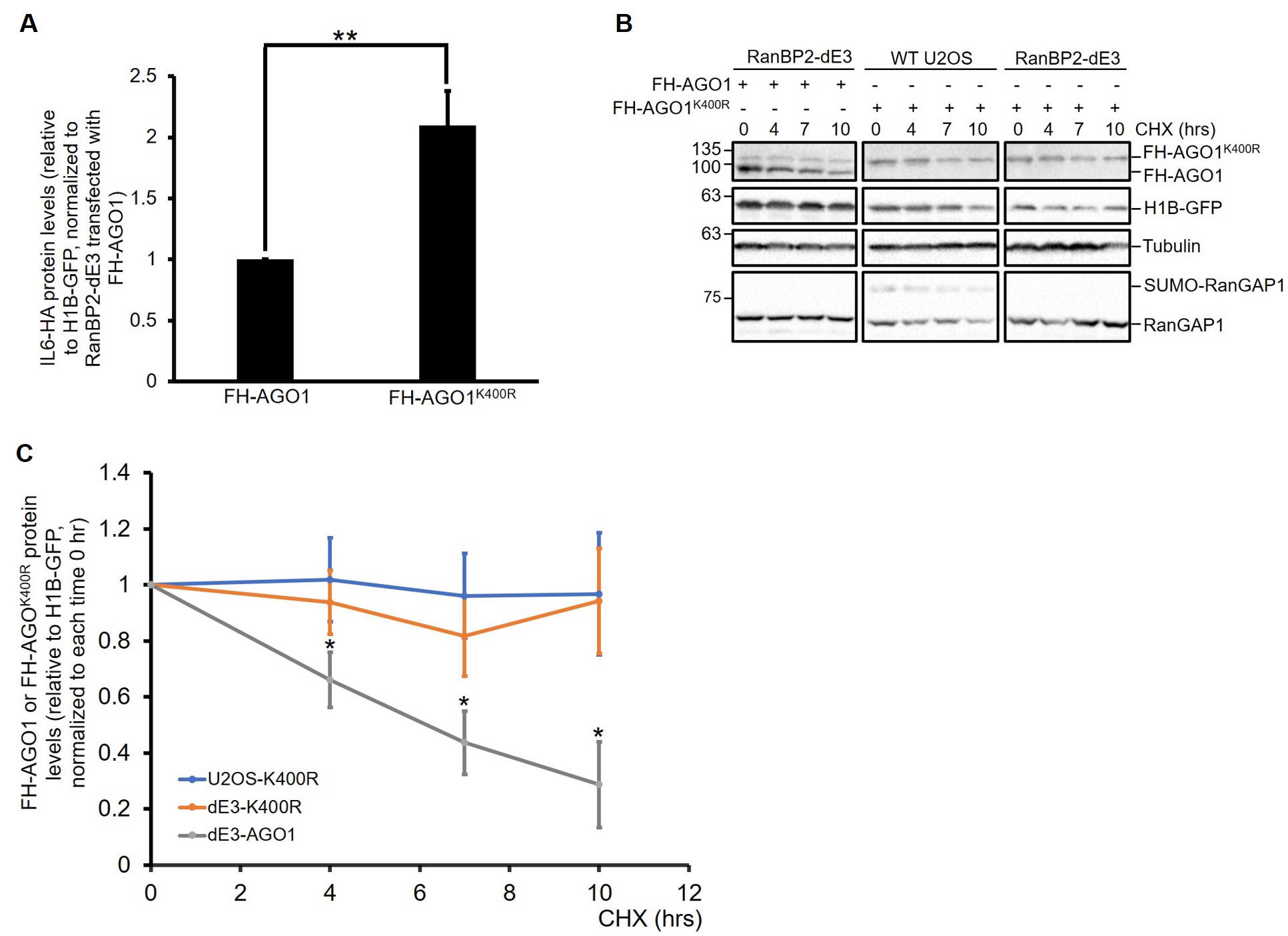

Supplement: S11 Fig — (A) Densitometry signals for IL6-HA and H1B-GFP in RanBP2-dE3 U2OS cells expressing FH-AGO1 and FH-AGO1K400R (see Fig 7I) were quantified. The signal was normalized to cells expressing FH-AGO1. Each bar is the average of three independent experiments ± SEM. **P = 0.001–0.01 (Student’s t-test). (B-C) Unmodified and RanBP2-dE3 U2OS cells were transfected with FH-AGO1 and FH-AGO1K400R and 18 h post-transfection treated with cycloheximide (CHX) for the indicated times to determine the decay rates of the two forms of FH-AGO1. Cell lysates were collected, separated by SDS-PAGE, and immunoblotted with antibodies against HA, GFP, Tubulin and RanGAP1. Note that FH-AGO1 primarily migrates just below the 100 kDa marker, while the FH-AGO1K400R migrates between the 100 and 135 kDa markers (in agreement with Fig 7I). The levels of the HA and α-tubulin immunoblot signals were analyzed by densitometry analysis and the ratio of FH-AGO1/α-tubulin was normalized to the zero time point. Each point is the average of three independent experiments ± SEM.*P = 0.01–0.05 (Student’s t-test). (TIF) [file pgen.1009378.s011.tif]

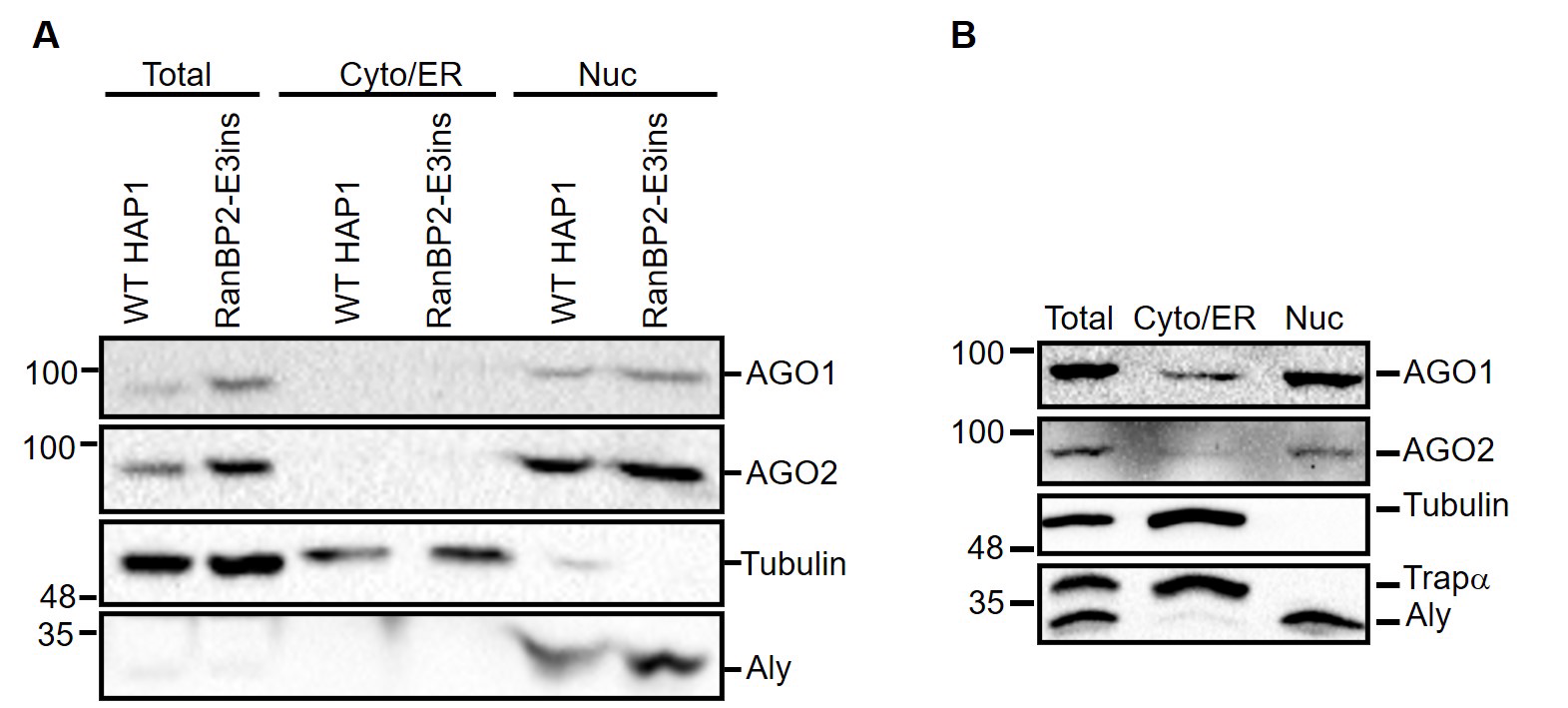

Supplement: S12 Fig — Total, Cytoplasmic/ER and nuclear fractions were isolated from unmodified and RanBP2-E3ins HAP1 cells (A) or HEK293 cells (B), separated by SDS-PAGE and immunoprobed for AGO1, AGO2, α-tubulin (cytosolic marker), Trapα (ER marker) and Aly (nuclear marker). Note that in all cell lines, the majority of the Argonaute proteins are nuclear. (TIF) [file pgen.1009378.s012.tif]

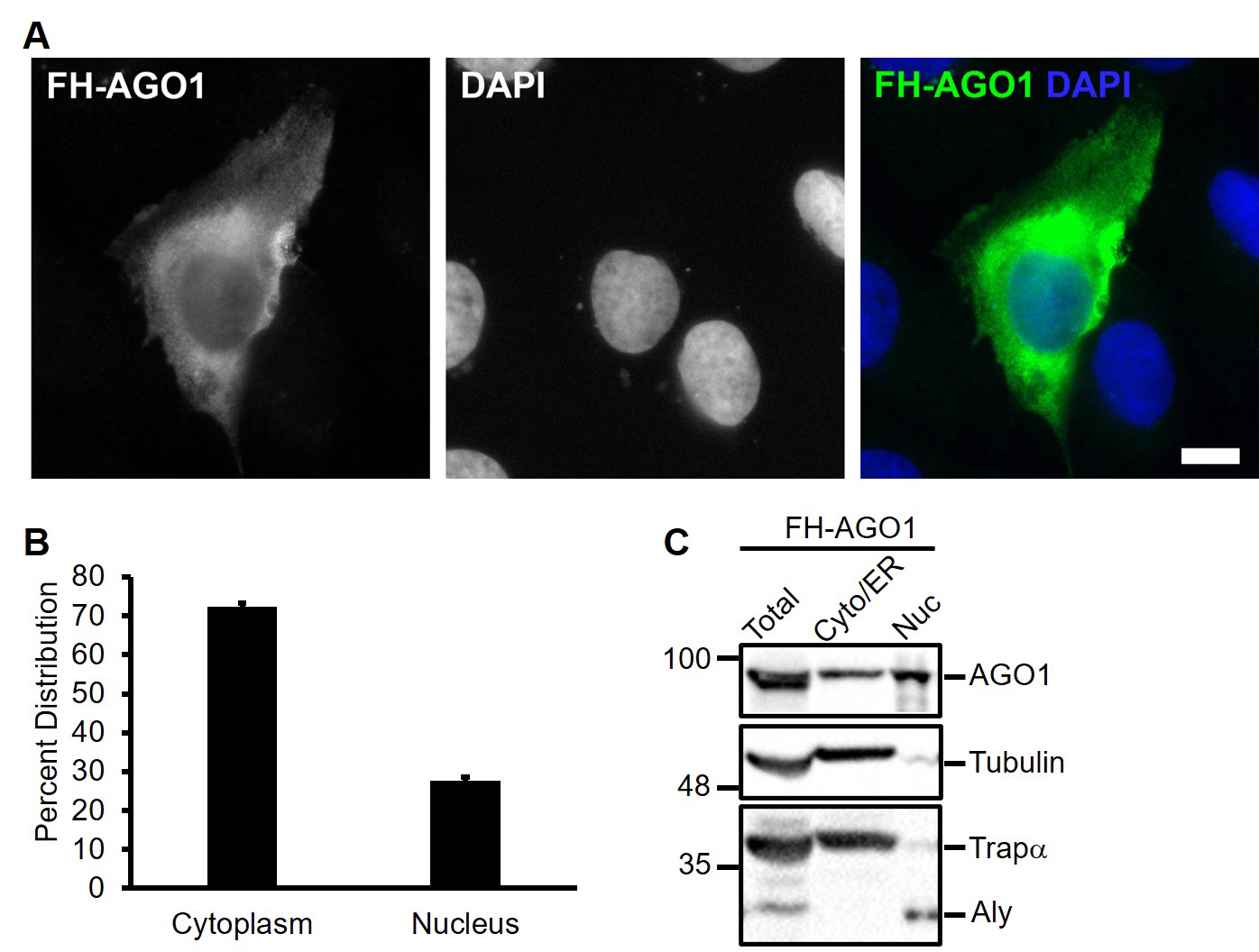

Supplement: S13 Fig — (A-B) U2OS cells were transfected with FH-AGO1 and after allowing expression for 18 hrs were fixed and immunostained for FLAG and DAPI stained to visualize DNA. Scale bar = 10 μm. A representative cell is shown (A) and the percent of the total integrated fluorescence in the cytoplasm and nucleus were quantified, each bar representing the average and standard error for 74 cells from two independent experiments. (C) Total, cytoplasmic/ER and nuclear fractions were isolated from U2OS cells that expressed FH-AGO1. Proteins from the lysates were separated by SDS-PAGE and immunoprobed for HA, α-tubulin (cytosolic marker), Trapα (ER marker) and Aly (nuclear marker). (TIF) [file pgen.1009378.s013.tif]
