# Supplementary material for: RanBP2/Nup358 enhances miRNA activity by sumoylating Argonautes
Source: PLoS Genet. 2021 Feb 18;17(2):e1009378. doi: 10.1371/journal.pgen.1009378 (PMC7924746; doi:10.1371/journal.pgen.1009378)
Supplement: S1 Table — (DOCX) [file pgen.1009378.s014.docx]

**S1 Table. ANE1-associated cytokines from case reports dating back to 1998**

| ANE1-associated cytokines | Number of 5’UTR Introns | Reference (Case reports that measured cytokine levels in patients) |
| --- | --- | --- |
| IL6 | None* | [1–8] |
| TNF-α | None | [1–3,7,8] |
| IL10 | None | [3,5,9] |
| IL15 | 2 | [5] |
| sTNFR1 | None** | [2–5] |
| IFNγ | None | [3,5] |
| IL1β | 1*** | [1,4,9] |
| IL2 | None | [9] |
| IL5 | None**** | [9] |

*- The major isoform lacks 5’UTR introns. There are three minor spliced isoforms, one that lacks a 5’UTR intron, one that uses an upstream transcriptional start site and whose extended 5’UTR has an intron, and a third that uses a downstream start codon and has a single 5’UTR intron.

**- The major isoform lacks 5’UTR introns. There are two spliced isoforms that contain the same 5’UTR but have been reported to use different start codons which are found in internal exons. These extended 5’UTRs have two and five introns, respectively.

***- There is a reported minor spliced isoform that has a different 5’UTR which also contains one intron.

****- The major isoform lacks 5’UTR introns. There are three reported minor spliced isoforms, one that lacks 5’UTR introns, and the remaining two with a single 5’UTR intron.

**References:**

1. Ichiyama T, Nishikawa M, Yoshitomi T, Hayashi T, Furukawa S. Tumor necrosis factor-alpha, interleukin-1 beta, and interleukin-6 in cerebrospinal fluid from children with prolonged febrile seizures. Comparison with acute encephalitis/encephalopathy. Neurology. 1998;50: 407–411.

2. Ichiyama T, Isumi H, Ozawa H, Matsubara T, Morishima T, Furukawa S. Cerebrospinal fluid and serum levels of cytokines and soluble tumor necrosis factor receptor in influenza virus-associated encephalopathy. Scand J Infect Dis. 2003;35: 59–61.

3. Ichiyama T, Endo S, Kaneko M, Isumi H, Matsubara T, Furukawa S. Serum cytokine concentrations of influenza-associated acute necrotizing encephalopathy. Pediatr Int. 2003;45: 734–736.

4. Ito Y, Ichiyama T, Kimura H, Shibata M, Ishiwada N, Kuroki H, et al. Detection of influenza virus RNA by reverse transcription-PCR and proinflammatory cytokines in influenza-virus-associated encephalopathy. J Med Virol. 1999;58: 420–425.

5. Kubo T, Sato K, Kobayashi D, Motegi A, Kobayashi O, Takeshita S, et al. A case of HHV-6 associated acute necrotizing encephalopathy with increase of CD56bright NKcells. Scand J Infect Dis. 2006;38: 1122–1125. doi:10.1080/00365540600740520

6. Kawada J, Kimura H, Ito Y, Hara S, Iriyama M, Yoshikawa T, et al. Systemic cytokine responses in patients with influenza-associated encephalopathy. J Infect Dis. 2003;188: 690–698. doi:10.1086/377101

7. Akiyoshi K, Hamada Y, Yamada H, Kojo M, Izumi T. Acute necrotizing encephalopathy associated with hemophagocytic syndrome. Pediatr Neurol. 2006;34: 315–318. doi:10.1016/j.pediatrneurol.2005.08.030

8. Tabarki B, Thabet F, Al Shafi S, Al Adwani N, Chehab M, Al Shahwan S. Acute necrotizing encephalopathy associated with enterovirus infection. Brain Dev. 2013;35: 454–457. doi:10.1016/j.braindev.2012.07.001

9. Kansagra SM, Gallentine WB. Cytokine storm of acute necrotizing encephalopathy. Pediatr Neurol. 2011;45: 400–402. doi:10.1016/j.pediatrneurol.2011.09.007
