# Supplementary material for: RanBP2/Nup358 enhances miRNA activity by sumoylating Argonautes
Source: PLoS Genet. 2021 Feb 18;17(2):e1009378. doi: 10.1371/journal.pgen.1009378 (PMC7924746; doi:10.1371/journal.pgen.1009378)
Supplement: S1 Text — RanBP2-mediated translation inhibition of the IL6-HA reporter requires cis-elements in both the IL6 5′UTR and 3′UTRs. (DOC) [file pgen.1009378.s015.doc]

**Supplementary Text**

**Most ANE1-associated cytokine genes have SSCRs that contain adenines and have low 5IMP scores**

Previously, we found that RanBP2 potentiates the translation of mRNAs that contain adenine-depleted signal sequence coding regions (SSCRs) at the beginning of their ORF [1]. Indeed these SSCRs tend to have long tracts of adenine-depleted sequence [2]. Moreover, genes that contain adenine-depleted SSCRs tend to lack introns in their 5′UTRs, when compared to other genes in the human genome [3]. As a result, there are typically no introns upstream of adenine-depleted SSCRs, thus placing these elements within the first exon at rates that are higher than expected [3]. Genes that contain adenine-depleted SSCRs are also associated with a variety of other features in the 5′ end of the ORF (e.g., presence of certain GC-rich motifs, enhanced presence of N1-methyladenosine), which are also associated with a lack of introns in their 5′UTR [4]. Previously, we used machine learning to evaluate the 5′ end of ORFs for these features which are summed up into a 5′UTR intron minus prediction (5IMP) score [4].

Since ANE1-associated cytokines (see S1 Table) are produced from mRNAs that contain SSCRs, we decided to evaluate whether they are also depleted of adenines and have high 5IMP scores. Of note, most of these mRNAs are produced from genes that lack introns in their 5′UTRs (S1 Table). Despite this, almost all ANE1-associated cytokines had very small adenine-less tracts when compared to other SSCR-containing genes that lacked introns in their 5′UTRs (“5UI-”) from the human genome (S1A Fig). Indeed, the length of their longest adenine-less tract was on par with non-SSCR containing genes and intergenic sequences (S1A Fig), strongly indicating that there is no selection for adenine-depletion in most ANE1-associated cytokine genes. One exception was IL6, which had a relatively long adenine-less tract. When 5IMP scores were evaluated, all ANE1-associated cytokines, including IL6, had low scores (<5) when compared to other SSCR-containing genes that lack 5′UTR introns (“SSCR 5UI-”) where a significant fraction have scores greater than 7 (S1B Fig).

From these results we conclude that the sequence composition of SSCRs from ANE1-associated cytokine mRNAs do not have the features that are normally associated with mRNAs whose translation is upregulated by RanBP2.

**RanBP2-mediated translation inhibition of the *IL6-HA* reporter requires cis-elements in both the *IL6* 5′UTR and 3′UTRs**

To determine the *cis*-elements in the human *IL6* mRNA that were responsible for RanBP2-dependent regulation, we created a series of chimeric and deletion reporters, transfected these in control and RanBP2-depleted cells and assessed reporter protein levels by immunoblot. Previously we demonstrated that the SSCR conferred RanBP2-dependent regulation on mRNAs encoding secretory proteins [1]. Notably the IL6 SSCR did not appear to have features present in SSCRs that are regulated by RanBP2, like high 5IMP scores (S1B Fig). Replacing the *IL6* SSCR with the *major histocompatibility complex* (*MHC*) SSCR (S6A Fig), which promotes translation in a RanBP2-dependent manner [1], did not affect RanBP2-dependent silencing (S6B-C Fig). In contrast, replacing either the *IL6* 5′UTR or the 3′UTR sequences within the *IL6-1i* construct with the corresponding sequences from *ftz* (to form *5F-IL6-1i*-5′UTR swapped, and *IL6-1i-3F*- 3′UTR swapped; S6D Fig)*,* disrupted RanBP2-dependent silencing (S6E-F Fig). Interestingly, the 5′UTR swapped construct produced very little protein in both control and RanBP2-depleted cells and this was validated when we swapped the 5′UTR with that of the human *β-globin* gene (to form the *5βG-IL6-1i* construct - S6D Fig; protein levels shown in S6G-H Fig). Thus, there appears to be multiple elements that are required for RanBP2-dependent silencing.

Next, we decided to characterize the 3′UTR element RanBP2-responsive element. We found that *IL6-1i-3del1,* which lacks the first 110 nucleotides of the IL6 3′UTR (S6I Fig), was still regulated by RanBP2 (S6J-K Fig), while *IL6-1i-3del2*, which lacks the last 317 nucleotides (S6I Fig), was no longer regulated by RanBP2 (S6J-K Fig). We noted that this deletion eliminated a *Let7a* miRNA binding site.

From these results we concluded that there was at least one RanBP2-dependent *cis*-element in the later part of the 3′UTR. It is also possible that there is a second *cis*-element in the 5′UTR, although we cannot rule out the possibility that in the absence of this element, so little IL6 protein is made that its downregulation by RanBP2 is hard to detect.

**References:**

1. Mahadevan K, Zhang H, Akef A, Cui XA, Gueroussov S, Cenik C, et al. RanBP2/Nup358 potentiates the translation of a subset of mRNAs encoding secretory proteins. PLoS Biol. 2013;11: e1001545. doi:10.1371/journal.pbio.1001545

2. Palazzo AF, Springer M, Shibata Y, Lee C-S, Dias AP, Rapoport TA. The signal sequence coding region promotes nuclear export of mRNA. PLoS Biol. 2007;5: e322. doi:10.1371/journal.pbio.0050322

3. Cenik C, Chua HN, Zhang H, Tarnawsky S, Akef A, Derti A, et al. Genome analysis reveals interplay between 5’UTR introns and nuclear mRNA export for secretory and mitochondrial genes. PLoS Genetics. 2011;7: e1001366. doi:10.1371/journal.pgen.1001366

4. Cenik C, Chua HN, Singh G, Akef A, Snyder MP, Palazzo AF, et al. A common class of transcripts with 5’-intron depletion, distinct early coding sequence features, and N(1)-methyladenosine modification. RNA. 2017;23: 270–283. doi:10.1261/rna.059105.116
